# Loss of the proteasomal deubiquitinase USP14 induces growth defects and a senescence phenotype in colorectal cancer cells

**DOI:** 10.1038/s41598-024-63791-5

**Published:** 2024-06-06

**Authors:** Johannes Gubat, Linda Sjöstrand, Karthik Selvaraju, Kübra Telli, Pádraig D’Arcy

**Affiliations:** Department of Biomedical and Clinical Sciences, Linköping University, 581 83 Linköping, Sweden

**Keywords:** Cell growth, Ubiquitylation, Senescence, Deubiquitylating enzymes, Target validation

## Abstract

The proteasome-associated deubiquitinase USP14 is a potential drug target. Using an inducible USP14 knockout system in colon cancer cells, we found that USP14 depletion impedes cellular proliferation, induces cell cycle arrest, and leads to a senescence-like phenotype. Transcriptomic analysis revealed altered gene expression related to cell division and cellular differentiation. USP14 knockout cells also exhibited changes in morphology, actin distribution, and expression of actin cytoskeletal components. Increased ubiquitin turnover was observed, offset by upregulation of polyubiquitin genes *UBB* and *UBC*. Pharmacological inhibition of USP14 with IU1 increased ubiquitin turnover but did not affect cellular growth or morphology. BioGRID data identified USP14 interactors linked to actin cytoskeleton remodeling, DNA damage repair, mRNA splicing, and translation. In conclusion, USP14 loss in colon cancer cells induces a transient quiescent cancer phenotype not replicated by pharmacologic inhibition of its deubiquitinating activity.

## Introduction

Malignant changes in cancer cells make them especially reliant on the cellular machinery for protein degradation. The demands of incessant growth caused by aberrant oncogenic signaling lead to a compensatory increase in protein synthesis to maintain cellular proteostasis. In addition, heightened genomic instability resulting from the loss of tumor suppression mechanisms can prompt the emergence of novel mutations, disrupting protein balance and fostering the production of aberrant proteins. This shift towards oncogenic signaling triggers an increased generation of misfolded proteins, posing a significant threat if left to accumulate. The Ubiquitin Proteasome System (UPS) is responsible for up to 80% of cellular protein degradation and constitutes a central component of the cellular response to oncogenic proteostasis. Consequently, various components of the UPS have been explored as potential drug targets^1^.

The UPS operates via a tagging mechanism directed by ubiquitin ligases, which mark damaged or short-lived proteins for destruction with the highly conserved small protein ubiquitin. Proteins tagged with K48-linked ubiquitin chains are guided to the 26S proteasome, a large multiprotein complex that functions as the cell’s molecular shredder. The 26S proteasome comprises the 19S regulatory particle (19S RP) and the 20S core particle (20S CP). The 19S RP recognizes ubiquitinated substrates via specific receptors, leading to deubiquitination and unfolding followed by translocation into the 20S CP where proteolysis takes place^2^.

The human genome encodes for 79 putative functional deubiquitinase (DUB) enzymes^3^, three of which are associated with the proteasome. USP14 and UCHL5 are cysteine-based DUBs which associate with the 19S RP subunits PSMD2 and ADRM1 respectively, whereas PSMD14 is a metalloprotease and is an intrinsic subunit crucial for the stability of the 19S RP^4^. PSMD14 is positioned near the 20S CP entry port and deubiquitinates the substrate as it is being translocated, thereby avoiding the translocation of ubiquitin^5,6^. The two cysteine DUBs regulate proteasomal activity by trimming ubiquitin chains. UCHL5 was demonstrated to rapidly debranch polyubiquitin chains while USP14 has been shown to remove supernumerary polyubiquitin chains en bloc. Inactivation of USP14 or UCHL5 causes different sets of ubiquitinated proteins to accumulate and thus have selective effects on protein degradation^7–9^. Additionally, USP14 has been shown to both inhibit and promote different proteasomal activities through an allosteric mechanism influenced by ubiquitin binding^10–12^.

USP14 has emerged as an interesting target for cancer and neurodegenerative disorders. USP14 regulates key proteins involved in critical cellular processes, including cell cycle (Cyclin B1, Aurora kinase B), autophagy (UVRAG), and has been shown essential for murine neuromuscular development by maintaining the free ubiquitin pool^13–16^. Several studies have shown a role for USP14 canonical Wnt signaling, acting as a positive regulator by deubiquitinating Dishevelled (Dvl). Interestingly, USP14 expression correlates with Wnt activation in colorectal and hepatocellular cancer tissues. In cellular models of hormone-responsive tumors—such as breast, ovarian, and prostate cancer—USP14 overexpression is correlated with increased cell proliferation. Importantly, both pharmacologic and RNA-interference-based inhibition of USP14 have shown efficacy in reducing cellular proliferation in prostate and breast cancer cells, as well as enhancing the anti-cancer effects of the androgen receptor (AR) inhibitor, enzalutamide^17–22^.

Selective and reversible inhibitors of USP14, such as the IU1 series of compounds, have been developed as potential therapies. These compounds enhance the degradation of proteasomal substrates, including Tau implicated in Alzheimer's disease. IU1 inhibitors also exhibit potential in modulating cancer-related processes like autophagy, DNA damage response, and AR signaling, suggesting their potential as adjuncts to standard cancer treatments^20,23,24^. The dienone-based compound b-AP15 inhibits both USP14 and UCHL5, demonstrating cytotoxicity in various cancer models, including Ewing’s sarcoma, bortezomib-resistant multiple myeloma, and mantle cell lymphoma. However, recent findings indicate that while USP14 inhibition contributes to b-AP15 cytotoxicity, other targets are involved. Despite the potential for USP14 inhibition in cancer therapy, our understanding of its effects remains limited, given the observed pleiotropy and its complex role in regulating cancer-related pathways^4,25–28^.

In this study, we aim to characterize the functional and molecular consequences of USP14 loss in colorectal cancer cells using an inducible knockout model. We identify a senescent phenotype associated with genetic loss of USP14, distinct from the effects of pharmacologic inhibition. By examining proteins physically associated with USP14 independently of the proteasome using available databases, our results offer new insights into USP14's functional role, with potential implications for the development of anti-cancer therapies.

## Results

### Loss of USP14 slows cell proliferation and alters the actin cytoskeleton

**Figure 1:**
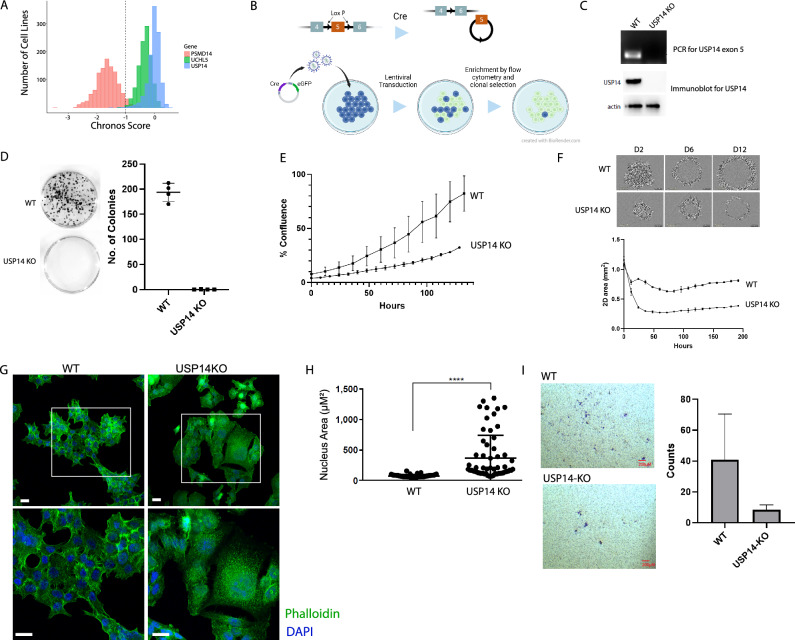
Functional consequences of USP14 loss. (**A**) Chronos scores for loss of proteasomal DUBs in DepMAP. The dashed line (− 1) represents the median Chronos score of reference essential genes. Reference non-essential genes have a median chronos score of 0. (**B**) Scheme for USP14 KO generation. (**C**) Validation of USP14 knockout after enrichment by flow cytometry. PCR and gel electrophoresis show the absence of *USP14* exon 5. USP14 is undetectable on immunoblotting. (**D**) Colony formation assay. Cells were harvested 72 h post-transduction and replated under puromycin selection. Colonies were stained 72 h post-transduction. The bars represent the average of four replicates ± SD. (**E**) Confluence of monolayer cultures of WT and USP14 KO cells measured over 130 h using live-cell imaging. Each point is the average of three replicates ± SD. (**F**) Growth of WT and USP14 KO spheroids. The line plot shows the 2D area of spheroids over time measured using live-cell imaging. Each point is the average of three replicates ± SD. (**G**) Confocal image showing differences in morphology and actin distribution between WT and USP14KO cells. Lower row of images show magnified versions corresponding to the white box on the top row images. White bar is 20 µM. (**H**) Nucleus area measured from confocal images. Bars represent the average, first and third quartiles of the nuclei measured (n = 62). (**I**) Boyden chamber assay. Representative images of the stained cells are shown on the left. Columns represent the average of number of stained cells counted from four microscopic fields in three samples ± SD.

We first examined the essentiality of the three proteasome-associated DUBs in cancer cell lines using data derived from pooled CRISPR/Cas9 dropout screens in the Cancer Dependency Map (DepMap) database. Depletion or expansion of cells following CRISPR/Cas9 mediated knockout are reported as Chronos scores which are scaled so that the median score of reference essential genes is − 1 and nonessential genes is 0^29–32^. In agreement with previous studies^33,34^, only PSMD14 is essential among the proteasome-associated DUBs in most of the cell lines (Fig. 1A).

To gain more insight into USP14 function, we investigated the early sequelae of USP14 loss including transient effects on cellular proliferation and effects that do not directly influence cellular viability which might not be apparent in the pooled CRISPR screens in DepMap. We developed a conditional USP14 knockout system in the colorectal cancer cell line HCT116 (Fig. 1B). In this system, exon 5 of *USP14* is flanked by Lox-P sites allowing for controlled deletion following expression of Cre recombinase. Transduction with Cre-recombinase and enrichment of transduced cells using fluorescence-activated cell sorting (FACS) resulted in a USP14 knockout population (referred to as USP14 KO) where *USP14* exon 5 is lost and the USP14 protein is undetectable on immunoblotting (Fig. 1C). A parallel transduction with GFP and puromycin resistance served as a control (referred to as WT). In a clonogenic assay, the USP14 KO cells failed to form visible colonies after 14 days, suggesting a growth defect when grown at low density in puromycin-containing medium (Fig. 1D). However, we could establish stably growing USP14 KO cell lines through limiting dilution from the FACS sorted cells but over significantly longer time frames (~ 2 months). The slow growth of the knockouts in the sparse cultures may indicate a reduced ability to produce required mitogens.

To measure cell proliferation and document changes in cellular morphology, we used cells < 10 passages after cell sorting. The USP14 KOs showed a significantly slower proliferation rate compared to WT cells (Fig. 1E). This difference is also observed in a spheroid formation assay where USP14 KOs grow slower and retain a smaller spheroid size (Fig. 1F). Similarly, siRNA-induced inhibition of USP14 in HCT116 cells, as well as in other colorectal cancer cell lines DLD1 and SW620 decreased cell proliferation (Supplementary Fig. S1). Overall, the loss of USP14, while tolerated, impairs cellular proliferation.

The USP14 KO cells displayed noticeable morphological changes, such as increased cell size and enlarged nuclei (Fig. 1G,H). Additionally, cytosolic actin staining revealed a less organized pattern in contrast to the predominantly cortical distribution and prominent lamellipodia observed in WT cells. Similar changes in actin distribution have been reported in previous studies after USP14 knockout suggesting a potential role in cytoskeleton remodeling^34^. We investigated whether the cell’s ability to migrate through a Boyden chamber assay using 10% FBS as a chemoattractant. We found that USP14 KO cells reduced ability to migrate compared to WT cells (Fig. 1I). Our findings are corroborated in a zebrafish model where USP14 KO cells form smaller tumors and disseminate less compared with WT cells (Supplementary Fig. S2).

### Changes in the transcriptome following USP14 loss

**Figure 2:**
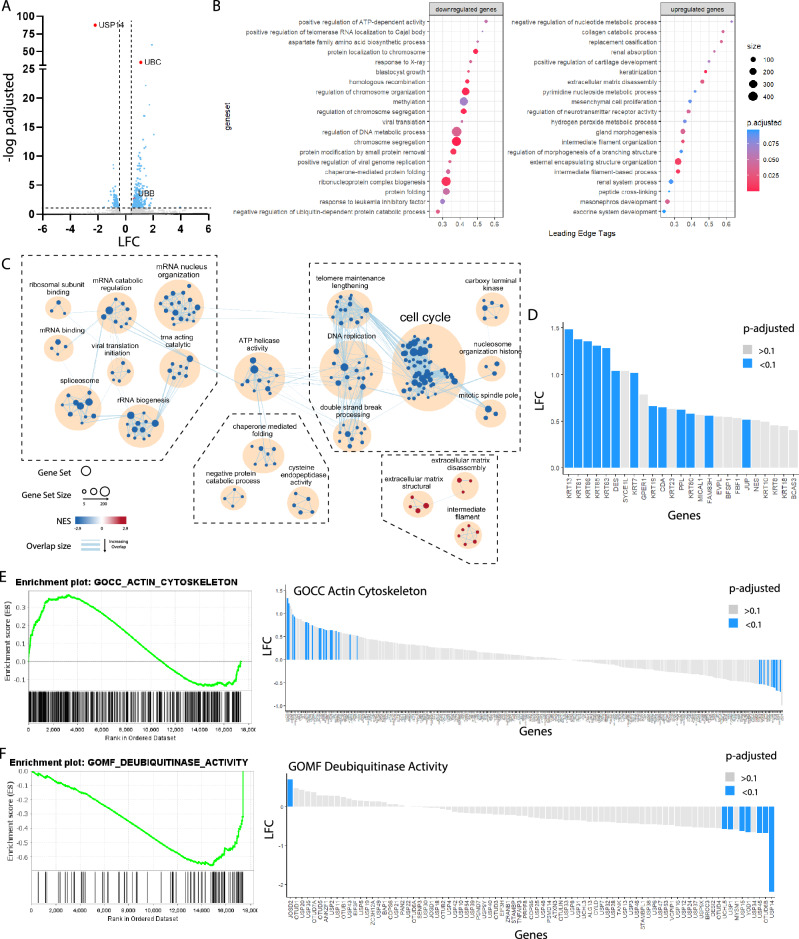
Transcriptomic analysis of USP14 KO cells. (**A**) Differential gene expression of USP14KO vs WT based on RNAseq. Points represent individual genes. Those in blue have a p.adjusted of < 0.1. Marked in red are *USP14* the polyubiquitin genes *UBB* and *UBC*. (**B**) Non-redundant GO terms identified using REVIGO that were the most over-represented in upregulated or downregulated genes. Leading Edge Tags represent the fraction of the genes in the RNAseq found in the gene set that contributes to the enrichment score. (**C**) Enrichment map of significantly enriched gene sets showing overarching themes. For the downregulated genes, over-represented GO terms are related to the cell cycle, RNA processing, and protein recycling. GO terms over-represented in upregulated genes are related to the extracellular matrix and intermediate filament modification. (**D**) Commonly upregulated genes in GO terms related to intermediate filaments. Bars are colored blue based on adjusted p-values to highlight the proportion of genes that are differentially expressed. (**E**) Enrichment plot and differential expression of component genes in the gene set GOCC actin cytoskeleton. (**F**) Enrichment plot and differential expression of component genes in the gene set GOMF DUB activity enrichment.

To further characterize the effects of USP14 loss, we performed RNA sequencing on USP14 KO and WT controls 14 days post-transduction. Out of the 17,760 genes quantified, 499 (2.8%) genes were upregulated and 361 (2.0%) genes were downregulated (p.adjusted < 0.1), using a fold-change cutoff of ± 1.2. The complete list of differentially expressed genes can be found in Supplementary File 1. Interestingly, a significant upregulation of the inducible polyubiquitin genes *UBB* and *UBC* was observed in USP14 KO (Fig. 2A). These genes are normally stimulated in response to environmental insults such as oxidative stress (NRF2-mediated) or proteotoxic stress (HSF1-mediated)^35^, where the demand for ubiquitin is increased. Thus, the upregulation observed may serve as a compensatory mechanism to replenish decreased ubiquitin pools following loss of USP14.

We performed pre-ranked Gene Set Enrichment Analysis (GSEA) to identify over-represented Gene Ontology (GO) terms. We extracted representative GO terms using REVIGO^36^, and grouped these genesets according to themes identified using EnrichmentMap and AutoAnnotate apps in Cytoscape^37–39^. The top representative GO terms for upregulated genes were associated with extracellular matrix and intermediate filament modification, while downregulated genes included cell cycle, RNA processing, and protein recycling (Fig. 2B,C). On closer inspection, many of the upregulated gene sets are associated with tissue differentiation. We examined closely related gene sets encompassing intermediate filaments and identified 25 common genes that were upregulated. Among these, 8 different keratins associated with epithelial differentiation were significantly upregulated. Other upregulated genes include *PPL* and *JUP* which are involved in intercellular attachments in keratinocytes and *DES*, which codes for the muscle-specific protein Desmin (Supplementary Fig. S3, Fig. 2D)^40^. As a benchmark for changes in actin-associated gene expression, we used the genes under the GO term GOCC Actin Cytoskeleton which was also found over-represented in the upregulated genes (Fig. 2E). A significant fraction of the gene set is differentially regulated (p.adjusted < 0.1, 30/362 genes). The GOMF Deubiquitinase Activity gene set, which included most of the deubiquitinases, was significantly over-represented in the downregulated genes. We found that several DUBs were significantly downregulated (Fig. 2C,F) in the USP14 KO suggesting that its interaction with the other DUBs is more complex than shared deubiquitinating activity.

The observed changes in gene expression and DUB activity imply that loss of USP14 induces widespread alterations in cell behavior, possibly toward a more quiescent and differentiated phenotype. Moreover, the loss of USP14 impacts the gene expression of multiple DUBs.

### Loss of USP14 induces a senescence-like phenotype

**Figure 3:**
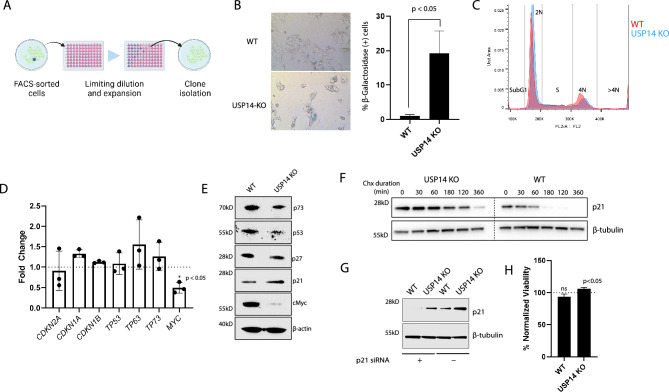
USP14 loss results in increased expression of β-Galactosidase associated with stabilization of p21. (**A**) Schematic for the isolation of pure USP14 KO clones. (**B**) β-Galactosidase staining. Representative photomicrographs of stained WT and USP14 KO cells are shown. Bars represent the average % β-Galactosidase positive cells ± SD from 15 microscopic fields. (**C**) Cell cycle analysis with Propidium Iodide and BrdU staining. An overlay of the histograms at the FL2 channel for WT and USP14 KO cells. (**D**) Fold change of select cell cycle regulators based on qPCR. Bars represent the average fold change of three replicates ± SD. *MYC* was significantly downregulated in the knockouts compared to the control based on a two-tailed paired T-test of dCT values (p < 0.05). (**E**) Corresponding immunoblots for the cell cycle regulators. Baseline p63 could not be detected and is not included here. (**F**) Cycloheximide chase for p21 in WT and USP14 KO. The cells were exposed to 100 µg/mL cycloheximide and were prepared for p21 immunoblotting at the specified times. (**G**) Immunoblots for p21 48 h post-transfection with p21 siRNA or negative control. (**H**) Cell viability after siRNA-mediated silencing of p21, measured with the CellTiter-Glo^®^ assay. % Normalized Viability is the viability of p21-transfected cells normalized to the viability of cells transfected with negative control siRNA. Bars represent the average of three replicates ± SD. The viability of p21-transfected USP14 KO cells is significantly higher than its corresponding control based on a paired T-Test (p < 0.05). The uncropped blots for the images used in this figure may be found in Supplementary (Supplementary Fig. S6).

For the subsequent experiments, we used pure USP14 KO cells expanded from single cell colonies through limiting dilution which have been passaged < 20 times (Fig. 3A). Morphologic alterations similar to what we have observed in USP14 KO cells have been previously described in cells undergoing senescence^41^. To examine whether USP14 KO cells display a senescent phenotype, we stained for β-galactosidase (β-gal), which is known to accumulate in senescent cells^42^. We found a significantly higher number of β-gal (+) cells in the USP14-KO compared to the control at < 10 passages (Fig. 3B). On subsequent passaging, we observed a decrease in β-galactosidase staining and a concomitant decrease in the gap in proliferation rates reflecting dropout of potentially senescent cells and selection of fitter clones (Supplementary Fig. S4). Cell cycle analysis revealed a higher 2N fraction in the USP14 KO which indicates either a G1 arrest or more cells going into G0 (Fig. 3C).

We next examined the gene and protein expression of various tumor suppressors implicated in senescence, including cyclin-dependent kinase inhibitors, *TP53*, and the proto-oncogene *MYC*. Apart from *MYC*, we observed no significant changes at the mRNA level (Fig. 3D). *MYC* was significantly decreased in the USP14 KO cells at the mRNA and the protein level (Fig. 3E). Additionally, we observed an increase in p21 and a decrease in p53 proteins in USP14 KO cells (Fig. 3E). p53 is known to directly induce the expression of p21 to effect cell cycle arrest as part of the response to genomic damage^43^. A previous study on *Tp53*(−/+) mice demonstrated that inhibition of USP14 through shRNA-mediated knockdown or by using the inhibitor IU1 increases p53 and p21 levels^44^. However, the observed increase in p21 levels in our study does not appear to be attributed to enhanced transcription or increased protein levels of p53 (Fig. 3D,E). In DLD-1 cells that have inactivated *TP53*^45^, p21 is also increased after siRNA-mediated knockdown of USP14 (Supplementary Fig. S5). We hypothesized that p21 stabilization is occurring upon USP14 KO, possibly due to alterations in ubiquitination, given its well-established status as a target for various E3 ligases and its vulnerability to ubiquitin-dependent proteasomal degradation^46^. To measure p21 degradation, we performed a cycloheximide chase experiment measuring protein levels through immunoblotting at various time points on cells exposed to cycloheximide. We found that p21 was stabilized in the USP14 KO cells (Fig. 3F). To examine if the increased levels of p21 affected the viability of the USP14 KO cells, we transfected the USP14 KO and WT cells with siRNA targeting p21 or non-targeting siRNA control and then measured the cellular viability after 108 h using Cell-Titer Glo^®^. At 48 h post-transfection, p21 levels are reduced to < 50% compared to control (Fig. 3G). The viability of USP14 KO cells was increased when p21 was inhibited compared to control, whereas it was unchanged in the WT cells (Fig. 3H). This suggests that p21 is involved in the slowing of cellular proliferation observed.

### Concomitant ablation of USP14 and UCHL5 strongly inhibits cell proliferation

**Figure 4:**
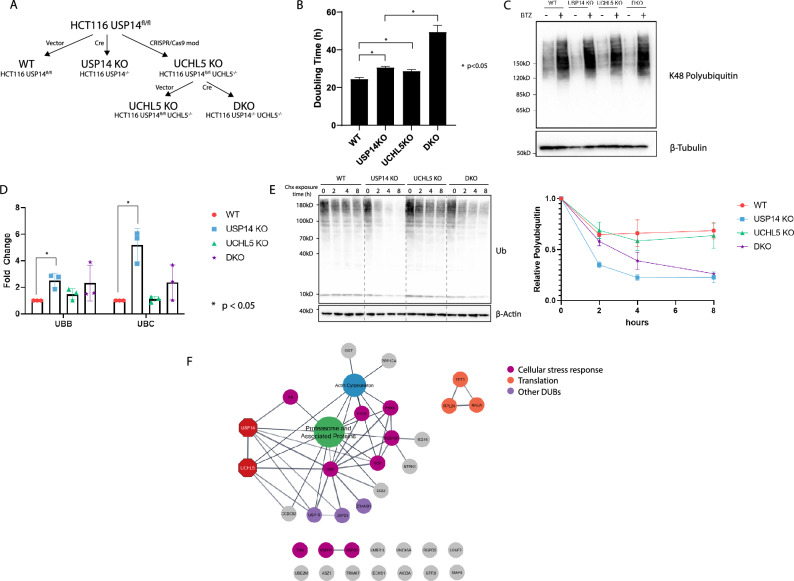
Effect of simultaneously knocking out USP14 and UCHL5 on cellular proliferation and ubiquitin turnover. (**A**) Scheme for the generation of the USP14, UCHL5 and USP14/UCHL5 double knockouts. (**B**) Doubling times of the knockouts. Cells were plated, expanded for 96 h and were counted. Doubling times are calculated based on monoexponential growth. Bars represent the average of three replicates ± SD. (**C**) Knockouts were exposed to 100 nM bortezomib or an equal amount of DMSO. Cells were lysed after 6 h for immunoblotting and probed for K48 polyubiquitin. (**D**) Differential expression of the inducible polyubiquitin genes *UBB* and *UBC* for the different knockouts based on qPCR. The bar plots represent the average fold change of three replicates ± SD. (**E**) The knockouts were exposed to 100 µg/mL cycloheximide for 2, 4, and 8 h and were probed for total ubiquitin. The amount of polyubiquitin was measured by densitometry and then normalized to the signal of its corresponding β-actin control. This is then normalized to the value at T0 to obtain the relative polyubiquitin in the samples. The points represent the average of three replicates ± SD. (**F**) Network analysis of common UCHL5 and USP14 interactors based on strings.db. Edges are based on high-confidence physical or functional interaction. Nodes that are known proteasome or actin cytoskeleton components are collapsed for simplicity. The full network is available in Supplementary (Supplementary Fig. S7). The uncropped blots for the images used in this figure may be found in Supplementary (Supplementary Fig. S8).

As UCHL5 also trims ubiquitin chains from proteasomal substrates, we hypothesized that its activity might allow cells to compensate for the loss of USP14. To investigate this, UCHL5 knockouts and USP14/UCHL5 double knockouts (UCHL5 KO and DKO hereafter) were generated from HCT116 cells bearing floxed *USP14*. UCHL5 KO cells were made by CRISPR/Cas9-editing and from this, DKOs were made using Cre/GFP transduction followed by FACS enrichment and expansion from single-cell clones (Fig. 4A). Measurement of proliferation rates was done on cells < 20 passages from the time the clones were isolated. Both USP14-KO and UCHL5-KO cells exhibited slower proliferation (doubling times of 30.6 and 28.7 h, respectively) compared to GFP controls (doubling time 24.5 h) (Fig. 4B). The doubling time of DKO cells was considerably longer (49.4 h) than the control and both knockouts, suggesting that the two DUBs possibly have an essential functional interaction.

Previous evidence demonstrated that polyubiquitin does not accumulate in cells lacking both USP14 and UCHL5^34^. Our knockouts confirmed this, and treatment with bortezomib resulted in a comparable accumulation of polyubiquitinated proteins. This suggests that the proteasome can sufficiently clear polyubiquitin conjugates even in the absence of the cysteine DUBs (Fig. 4C). We further assessed the impact of proteasomal DUBs on ubiquitin levels. While baseline total ubiquitin levels were similar across the knockouts (Fig. 4C), *UBB* and *UBC* expression was significantly upregulated in the USP14 KOs while the DKO trends towards an increase although not statistically significant (Fig. 4D). This suggests there is a corresponding increase in ubiquitin disposal in the USP14 KO and possibly in DKO as well. Using densitometric measures of total ubiquitin levels in a cycloheximide chase experiment, we observed a drop and plateau in total ubiquitin levels two hours after cycloheximide treatment, with a greater reduction in polyubiquitin in USP14 and DKOs compared to UCHL5 and WT, which exhibit similar patterns (Fig. 4E). Despite a potential functional interaction between USP14 and UCHL5, it does not appear to be linked to proteasome inhibition or ubiquitin depletion.

We explored possible links between the two DUBs through common physical interactors identified from curated experiments using the BioGRID protein interaction database^47^. To resolve direct and indirect interactions (interactors through connections with common complexes), we used strings.db^48^ to generate an interaction network and group the proteins into their associated complexes (Fig. 4F). However, we found no evidence of physical interaction between USP14 and UCHL5 outside the proteasome.

### Pharmacologic inhibition of USP14 with IU1 enhances ubiquitin turnover without inhibiting cell proliferation

**Figure 5:**
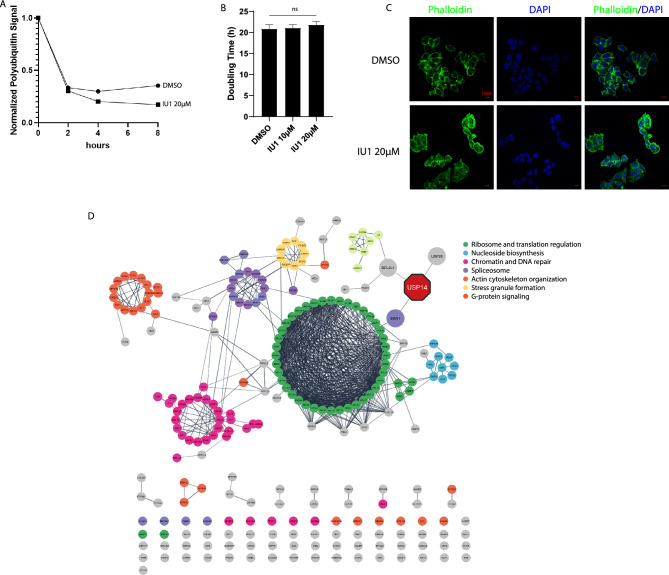
Pharmacologic inhibition of USP14 by IU1 and proteasome-independent USP14 interactome. (**A**) The cells were pretreated with 20 µM IU1 or DMSO, exposed to 100 µg/mL cycloheximide for the specified times, and then probed for ubiquitin. The amount of polyubiquitin was measured by densitometry and then normalized to the signal of its corresponding β-actin control. This is then normalized to the value at T0 to obtain the relative polyubiquitin in the samples. (**B**) Doubling time after 72 h exposure to DMSO, 10 µM or 20 µM IU1. Bars represent the average of three replicates ± SD. (**C**) Confocal image of cells after exposure to 20 µM IU1 for 72 h or DMSO. (**D**) Network analysis of the proteasome-independent interactome of USP14. Edges are high-confidence physical or functional interactions. Nodes are colored depending on the gene sets that they belong to after functional enrichment analysis. USP14 and its first-degree interactors are enlarged for emphasis.

We wanted to explore whether the observed effects on cellular phenotype after the knockout of USP14 could be recapitulated in cells treated with the USP14-specific inhibitor IU1^49^. IU1 has a documented IC50 of 4.7 µM^49^. In the WT cells, a 6-h treatment with 20 µM results decreased Ub-VS binding to USP14 (Supplementary Fig. S9). We pretreated WT cells with 20 µM IU1 and measured total ubiquitin levels in a cycloheximide chase experiment. Treatment with IU1 reduced the ubiquitin levels beyond two hours of cycloheximide exposure (Fig. 5A), similar to USP14 KO. We next tested the effect of IU1 on cellular proliferation. To simulate the long-term absence of USP14 activity in the USP14 KO cells, we cultured WT cells in the presence of 10 µM or 20 µM IU1 and calculated doubling times over 72 h using standard cell counting. At these doses, IU1 did not affect proliferation rates (Fig. 5B). We also did not observe the morphologic changes we previously found in the USP14 KO in the IU1-treated cells (Fig. 5C). Thus, while inhibition of the deubiquitinating activity of USP14 affects ubiquitin turnover, this does not appear to impact cellular proliferation or morphology. We considered the possibility that a higher dose of IU1 or its more potent analog, IU1-47, would be needed to impact cellular viability. We exposed cells to varying concentrations of IU1 or IU1-47 and measured viability using MTT assay. IU1 caused a modest decrease in viability at 100 µM without altering proteasome proteolytic activity, whereas IU1-47 started to affect viability at 10 µM. However, this effect was also observed in USP14 KO cells and is likely mediated by off-target effects at supra-pharmacologic concentrations (Supplementary Fig. S10).

A possible reason for the differences in outcomes between inhibition by IU1 and genetic loss are functions of USP14 outside the proteasome. To shed light on potentially novel roles of USP14, we identified other protein complexes that interact with USP14 which are not found to physically associate with proteasome subunits. We searched for known physical interactors of USP14 and selected 19S RP and 20S CP proteasome subunits (PSMA1, PSMA3, PSMA6, PSMB1, PSMB2, PSMB5, PSMD1, PSMD2, PSMD10, PSMD14, ADRM1) in BioGRID. We found a total of 1734 proteins interacting with the chosen proteasome subunits and 460 proteins interacting with USP14 (Supplementary Files 3 and 4). Among the 460 proteins that interact with USP14, 247 have not been shown to interact with the mentioned proteasome subunits. We grouped these 247 interactors by conducting a network and functional enrichment analysis using the stringApp plugin in Cytoscape to identify those which likely exist in a complex or are known to interact directly^50^. The resulting network primarily consists of proteins involved in the ribosome, chromatin, actin cytoskeleton, spliceosome, DNA repair, nucleoside biosynthesis, and stress granule formation (Fig. 5D). This supports the notion that USP14 participates in multiple cellular processes, possibly independently of the proteasome.

## Discussion

In this study, we employed functional studies, gene expression analysis, and protein interaction data to characterize the role of USP14 in cancer cells and assess its potential as a drug target. Using an inducible knockout model allowed us to observe the early phenotype following USP14 loss, capturing changes missed in stably dividing cells with established adaptations. In DepMap, it appears that loss of USP14 results only in a modest decrease in fitness. Transient or mild effects on cell proliferation may not be apparent in pooled CRISPR screens where the benchmark is a group of highly essential genes that have a large and direct effect on cellular viability. Our findings align with previous studies demonstrating USP14's role in regulating cancer cell proliferation^51–53^. While USP14 knockout cells remain viable, our results indicate a shift toward a more quiescent phenotype.

Transcriptomic changes reflect the downregulation of cell division genes, accompanied by a noteworthy upregulation of cytokeratin genes and cell junction proteins, suggesting a shift towards a more differentiated phenotype. Many of the USP14 KO cells initially exhibit a senescent phenotype. Over successive passages, this population is selected out, resulting in decreased β-galactosidase staining and increased proliferation rates, albeit remaining slower than the WT counterpart. We investigated the expression of proteins known to mediate senescence and among these, c-Myc, a master regulator of cell growth, was reduced in the USP14 KO cells while p21 was found to be stabilized. We found that siRNA-induced reduction of p21 results in an increase in proliferation only in USP14 KO cells. p21 is degraded by the proteasome after ubiquitination by the E3 ligases SCF/Skp2 and APC/C complex whose activity is tightly coupled with cell cycle events^46^. USP14 may affect one of these processes causing stabilization of p21. However, it could not be ruled out that the p21 increase is secondary to other events. While we have not pinpointed which proteins USP14 interacts directly with to effect these changes, it appears that p21 is one of the effectors.

The morphological changes we observed upon USP14 KO are likely associated with altered cytoskeleton behavior. A prior report showed increased actin, more stress fibers, and diminished intercellular contacts in USP14 KO, linked to tubulin downshifting to smaller structures relative to WT cells^34^. Interestingly, the USP14 interactome includes proteins that coordinate actin remodeling such as the Arp2/3 complex and cofilin family proteins, not found to be associated with the proteasome^54,55^. This suggests a role for USP14 in cytoskeletal organization, possibly independent of its role on the proteasome.

Our findings are in agreement with prior reports on USP14's role in maintaining ubiquitin levels^56–58^. Apart from the upregulation of polyubiquitin genes *UBB* and *UBC*, we noted significant shifts in the expression of other DUBs. Functional redundancy among DUBs implies a complex, interrelated network^59^, thus, changes in the expression of DUBs to accommodate perturbations in ubiquitin homeostasis may not at all be surprising. A previous study of the DUB landscape in breast cancer cells showed that USP14 is among the most abundant and active of DUBs^60^. Considering this, it is tempting to think that USP14 loss would be compensated for by an increase in other DUBs. Our results show, however, that when USP14 is knocked out, several DUBs are significantly downregulated. On the other hand, gene expression and DUB activity are not necessarily correlated. It is also possible that the changes in DUB expression address effects other than the lack of deubiquitinating activity.

While only USP14 is linked to polyubiquitin expression, UCHL5 seems poised to recover ubiquitin from proteasome-bound proteins. Loss of UCHL5, however, does not increase ubiquitin degradation. A previous study showed cell dependency on USP14 correlated with UBC, while dependency on UCHL5 correlated with members of the INO80 complex^61^. The interactomes suggest that these DUBs do not physically interact outside the proteasome complex, indicating independent functions. Intriguingly, cells knocked out for both DUBs exhibit a significant decrease in fitness, underscoring the potential of combined USP14-UCHL5 targeting.

Among the phenotypic changes that we observed in the USP14-KO, pharmacologic inhibition using IU1 reproduced the increase in ubiquitin degradation but had no effect on cellular proliferation and morphology. Considering the cell’s ability to modulate ubiquitin levels through various mechanisms^62^, the increased ubiquitin turnover might have little bearing on cellular viability under normal conditions. Under stress, the picture might be different. Previous studies have shown that loss of USP14 results in increased sensitivity to tunicamycin or DTT-induced proteotoxic stress, as well as sensitivity to cycloheximide exposure^34,56^. While we did not observe any effect on cellular proliferation, previous studies have shown that IU1 decreases cellular proliferation in different cancer cell lines although the concentrations at which this was observed varied widely—from as low as 0.2–100 µM^44,63,64^. Different cell lines may thus have varying susceptibilities to USP14 inhibition. In our model, losing *USP14* elicits a different phenotype from that of IU1 exposure. This suggests that USP14 can perform some of its functions despite inhibition of its catalytic activity on the proteasome.

Many investigations into the link between USP14 and cancer focus on specific protein targets, yet most of what is known about USP14 pertains to its proteasomal activity. This disparity underscores a knowledge gap regarding the broader roles of USP14 in the cell. A comparison of the USP14 and proteasome interactomes suggests that USP14 may function beyond its proteasomal activity. From a cancer therapeutics perspective, these extraproteasomal interactions may be relevant, introducing new possibilities for targets. However, questions arise about the actionable nature of these interactions. While loss of USP14 protein seems a more desirable objective for exerting anti-cancer effects, this is not simulated by pharmacologic inhibition. In the era of targeted protein degradation and PROTAC development, exploring this possibility is worth considering.

## Methods

### Cell culture and treatments

HCT116 cells (ATCC) were cultured in McCoy’s 5A (modified) medium supplemented with GlutaMAX™ (ThermoFisher), 10% fetal bovine serum (Gibco), and 100U penicillin–streptomycin (Gibco). The cells were maintained at 37 °C in a 5% CO_2_ humidified incubator and were regularly passaged at 70–80% confluence.

### Viral transduction and expansion of pure USP14 knockout cells

CRISPR-edited HCT116 with floxed *USP14* exon 5 (HCT116 USP14 flox) were plated at a density of 1 × 10^5^ cells/cm^2^ and incubated overnight. The cells were then transduced with either GFP-Cre or GFP lentiviral particles at a multiplicity of infection of 8, in medium containing 6 µg/µl Polybrene. After 48 h, fluorescence-activated cell sorting (FACS) with BD FACS Canto II (BD Biosciences) was done to separate GFP-expressing cells based on fluorescence on the FITC channel. To isolate pure USP14 knockout cells, the cells were serially diluted and were plated at a calculated number of 0.004–1000 cells per well on a 96-well plate using a 1:1 mix of conditioned medium obtained from the CRISPR-edited HCT116 and fresh culture medium. After 3 weeks, growing colonies were detached with trypsin and were subsequently expanded. Immunoblots for USP14 were done to verify the absence of USP14 in the generated mutants.

### Colony formation assay

HCT116 USP14 flox cells were transduced with Cre/GFP or GFP as described above. The cells were expanded, harvested after 72 h, and then plated into four 60 mm dishes at a density of 1000 cells per plate and cultured in the presence of 1 µg/ml puromycin for 14 days. Subsequently, colonies were fixed with 4% paraformaldehyde and stained with 0.5% crystal violet solution. After staining, the excess dye was removed by rinsing in water, and plates were air-dried before imaging and subsequent manual counting.

### Live-cell imaging for 2D cultures and spheroids

For 2D cultures, cells derived from stably expanding USP14 knockout clones were plated at a density of 1.7 × 10^4^ cells/cm^2^ on 96-well microplates. For spheroids formation, 4 × 10^3^ cells were plated on Costar^®^ Ultra-low attachment, 96-well plate. Serial phase-contrast images were taken with the IncuCyte ZOOM^®^ Live-cell analysis system (Essen Bioscience). Images were analyzed using the IncuCyte ZOOM^®^ software (Essen Bioscience) and area measurements were done in ImageJ.

### Boyden chamber assay

Millicell^®^ Transwell inserts with 12.0 µM pore size were set on a 12-well plate. The external compartment (between the well and the transwell) was filled with 1.5 mL growth medium supplemented with 10% FBS. The cells were resuspended in serum-free medium and plated onto the internal chamber (in the transwell). A total of 1 × 10^5^ cells were seeded per transwell along with 1 mL of serum-free medium. The setup was kept under standard growth conditions. After 24 h, the cells remaining in the internal chamber were removed with a cotton swab. The cells on the external chamber were fixed using methanol, stained with 0.5% crystal violet solution, and air-dried. The number of stained cells were counted from four contiguous and central 4 × fields taken using an inverted microscope.

### Long-term IU1 exposure

Stably expanding knockouts and their corresponding WT controls were grown in full medium supplemented with IU1 at 10 µM, 20 µM, or the corresponding amount of diluent (DMSO). 3 × 10^5^ cells were plated on T25 flasks, incubated, passaged, counted, and replated after 72 h.

### SDS-PAGE and immunoblotting

Cells were lysed using RIPA buffer, and lysates were prepared for electrophoresis with NuPAGE™ sample reducing agent (Thermofisher) and NuPAGE™ SDS loading buffer (Thermofisher). Samples were heated to 95 °C for 5 min. A total of 25 µg of protein was resolved with SDS-PAGE using 3–8% tris–acetate gels for blots probing polyubiquitin or 4–12% bis–tris gels for all the others. Proteins were transferred to nitrocellulose membranes using Thermo Scientific™ Pierce™ G2 Fast blot. Membranes were incubated in primary antibody diluted in PBST with 2% BSA overnight at 4 °C, washed, and incubated with the appropriate secondary antibody diluted in PBST with 5% skimmed milk. Blots were developed using Clarity Western ECL substrate (Bio-Rad) and imaged using ChemiDoc MP Imager (Biorad). Images were acquired with ImageLab v 5.2.1 (Biorad) using the built-in protocol “Chemi Hi-sensitivity” via autoexposure or manual exposure. Global contrast and gamma adjustments were done on the images. All antibodies used are in Supplementary Table 3.

### β-Galactosidase staining

HCT116 USP14 flox cells were transduced with Cre/GFP or GFP and enriched by flow cytometry as described above. The cells were then plated on glass slides mounted on tissue culture plates and were incubated at standard culture conditions for 48 h. Staining for β-Galactosidase was done using the β-Galactosidase Staining Kit (Abcam, Cat. ab102534) using manufacturer instructions. Photomicrographs were taken at 200 × magnification. β-Galactosidase positive and total cells were counted manually.

### Gene expression analysis

HCT116 USP14 flox cells were transduced with Cre/GFP or GFP and enriched by flow cytometry as described above. Triplicate samples were prepared and harvested for RNA extraction at D14 post-transduction. RNA was isolated using RNeasy Plus Mini Kit (Qiagen). The integrity and quantity of the starting material were determined by appropriate methods, e.g. volume and concentration measurement. Library preparation incorporated DNA fragmentation, adapter ligation, amplification, and size selection using proprietary methods of Eurofins Genomics. The reads were aligned to hg19/GRC37, UCSC with BowData v2.2.9 and Tophat 2.0.14. Count matrices were generated from .bam files with featureCounts and differential gene expression analysis was done using DESeq2 v1.40.2^65,66^. The p.values were based on a lfcThreshold parameter of 0.26303 (equivalent to fold change of ± 1.2). Analysis was performed using R 4.3.1 and RStudio 2023.09.0 + 463. Gene set enrichment analysis was done on GSEA 4.03 using a ranked list of genes based on the calculated t-statistic and Hallmarks and Gene Ontology as gene set databases. Network analysis was done using the EnrichmentMap and AutoAnnotate applications for Cytoscape 3.10.1. Enrichment map settings for Node Cutoff Q value < 0.05, and Edge Cutoff < 0.5. Some of the clusters were annotated manually for clarity^37–39^. Many of the GO terms are highly redundant, thus we extracted representative terms using REVIGO, an online tool that groups GO terms based on semantic similarity^36^.

### Cell fixation, staining, and cell cycle analysis

Cells derived from stably expanding USP14 knockout clones were exposed to 30 µM BrdU for 1 h at 60–80% confluence. The cells are then harvested from cultures with 0.25% Trypsin–EDTA and resuspended using the old medium where it was grown in. The cell suspensions were pelleted at 400 × G for 5 min, washed with PBS, and pelleted again. The resulting cell pellet was resuspended in 500 µL of PBS and was added dropwise into 5 mL of cold ethanol. The fixed cells were kept at − 20 °C for 72 h. After storage, the cells were washed two times by pelleting at 500 × *G* for 10 min and resuspended in PBS. A volume equivalent of 1 × 10^6^ cells was mixed with 0.5 mL 2N HCl/0.5% Triton X-100, incubated for 30 min, and neutralized with 0.1 M Na_2_B_4_O_7_. The cells were then resuspended in 100 µL of washing buffer (0.5% Tween 20/1% BSA in PBS) and incubated with 1 µg anti-BrdU antibody for 1 h. The cells were then pelleted, washed, resuspended in washing buffer, and incubated with Alexa fluor 488 anti-mouse at a 1:200 dilution. The cells were then pelleted, washed, and mixed with 500 µL of 20 µg/mL Propidium Iodide + 10 µg/mL RNase in PBS, and incubated in the dark at room temperature for 30 min. Flow cytometry was then done with Gallios™ Flow Cytometer (Beckman Coulter) and data was acquired using Kaluza Acquisition Software 1.0 (Beckman Coulter).

### Cycloheximide chase

The cells were plated at 1 × 10^5^ cells/well in 12-well plates and incubated for 16 h in complete medium. Cycloheximide (Sigma, Cat. 239765) was added to a working concentration of 100 µg/mL and the cells were harvested after the specified times. Untreated cells were used as 0 h controls. The protein lysates were then processed and probed for ubiquitin or p21 as described under *SDS-PAGE and Immunoblotting*.

### siRNA transfection

All of the siRNAs used in the study were procured from Qiagen (Supplementary Table 2). 37.5 ng siRNA was complexed with 3% HiPerFect transfection reagent (Qiagen, Cat. 301704) diluted in serum-free medium for 10 min in 24-well plates. 1.5 × 10^4^ cells suspended in 500 µL of complete medium were added into the transfecting solution. The cells were then incubated and maintained under standard culture conditions. Expression of target proteins was determined by immunoblotting 72 h post-transfection. Measurement of viability was done 108 h post-transfection with the CellTiter-Glo 2.0 assay (Promega) following the manufacturer's instructions.

### qPCR

Cells were grown to 60–70% confluence, washed and pelleted. RNA was isolated using RNeasy Plus Minikit (Qiagen) according to manufacturer instructions. cDNA synthesis and corresponding No-RT controls were made from 1 µg of RNA using iScript cDNA synthesis kit (Biorad). 50 µg of cDNA/No-RT control was diluted in RNAse-free water, mixed with the validated primers and SsoAdvanced Universal SYBR Green supermix (Biorad) in MicroAmp™ Fast Optical 96-Well Reaction Plates (ThermoFisher). qPCR was performed on the 7500 Fast Real-Time PCR system (Applied Biosystems) using the following thermal cycling protocol: 1. Activation (95C × 30 s) 2. Amplification (95C × 10 s > 60C × 30 s) for 40 cycles. Relative quantification was done on 7500 software v2.06 (Applied Biosystems) using ACTB as internal control. Selection of the internal control was made based on BestKeeper rank comparing multiple internal controls^67^. Information on the primers for targets and internal controls is available in Supplementary Table 1.

### Confocal microscopy

HCT116 cells were grown on coverslips until 60% confluent. Cells were fixed with 4% paraformaldehyde followed by permeabilization with 0.1% Triton X-100 and blocking with 2% BSA to prevent non-specific binding. To visualize actin filaments, cells were incubated with Alexa Fluor 488 Phalloidin (ThermoFisher). Coverslips were mounted on glass slides using ProLong Glass Antifade with NucBlue Stain (ThermoFisher) and cured for 24 h. Images were captured using LSM700 Zeiss.

### Generation of protein interaction network

Protein interaction networks were downloaded from BioGRID on October 10, 2023. Proteins of interest (proteasome-unassociated USP14 interactors and intersection of USP14 and UCHL5 interactors) were searched in the stringApp v2.0.1 plugin in Cytoscape v3.10.1, retaining only high-confidence interactions (0.700). Enrichment analysis was done in stringApp and node classifications were made based on the GO sets that the proteins belonged to.

### Statistical analysis

Data analysis was performed in RStudio 2023.09.0 + 463, R 4.3.1, and Microsoft Excel v2311. For qPCR data, the p-values are based on a two-tailed paired T-test using dCT values. CellTiter-Glo^®^ viability assay is based on a two-tailed paired t-test. All the other comparisons are based on two-tailed Student’s t-tests. All the tests are based on α = 0.05.

### Supplementary Information

Supplementary Information 1.

Supplementary Information 2.

Supplementary Information 3.

Supplementary Information 4.

Supplementary Information 5.

Supplementary Information 6.

Supplementary Information 7.

Supplementary Information 8.

## Data Availability

The RNAseq dataset has been deposited in GEO with the accession number GSE248258 (https://www.ncbi.nlm.nih.gov/geo/query/acc.cgi?acc=GSE248258).
