# Supplementary material for: Loss of the proteasomal deubiquitinase USP14 induces growth defects and a senescence phenotype in colorectal cancer cells
Source: Sci Rep. 2024 Jun 6;14:13037. doi: 10.1038/s41598-024-63791-5 (PMC11156967; doi:10.1038/s41598-024-63791-5)
Supplement: Supplementary file 1 — Supplementary Information 1. [file 41598_2024_63791_MOESM1_ESM.docx]

Qiagen RT2 PCR primers

1. Official Symbol

TP63 [Human]

Species

Human (Homo sapiens)

Entrez Gene Id

8626

Detected Transcripts

NM_001114978 (4833 bp)

Band Size

123 (NM_001114978),

Reference Position

770 (NM_001114978)

Official Name

glyceraldehyde-3-phosphate dehydrogenase

Official Symbol

1. GAPDH [Human]

Species

Human (Homo sapiens)

Entrez Gene Id

2597

Detected Transcripts

NM_001256799 (1455 bp), NM_002046 (1421 bp), NM_001289745 (1513 bp), NM_001289746 (1407 bp)

Bplength

1455

Band Size

89 (NM_001256799),89 (NM_002046),89 (NM_001289745),89 (NM_001289746),

Reference Position

1355 (NM_001256799)

1. RT² qPCR Primer Assay for Human 18SrRNA

(X03205). RT² qPCR Primer Assay

Biorad Prime PCR primers

1. **CDKN1A**

| **Technology:** |  | qPCR |
| --- | --- | --- |
| **Assay Type:** |  | SYBR® Green |
| **Application:** |  | Gene Expression |
| **Unique Assay ID:** |  | qHsaCID0014498 |
| **Assay Design:** |  | Intron-spanning |
| **Chromosome Location:** |  | 6:36652205-36653597 |
| **Amplicon Length:** |  | 159 |
| **Splice Variants Targeted:** |  | [ENST00000244741](http://www.ensembl.org/id/ENST00000244741) [ENST00000373711](http://www.ensembl.org/id/ENST00000373711) [ENST00000448526](http://www.ensembl.org/id/ENST00000448526)[ENST00000405375](http://www.ensembl.org/id/ENST00000405375) |

1. CDKN2A

| **Technology:** |  | qPCR |
| --- | --- | --- |
| **Assay Type:** |  | SYBR® Green |
| **Application:** |  | Gene Expression |
| **Unique Assay ID:** |  | qHsaCED0056722 |
| **Assay Design:** |  | exonic |
| **Chromosome Location:** |  | 9:21967834-21967949 |
| **Amplicon Length:** |  | 86 |
| **Splice Variants Targeted:** |  | [ENST00000304494](http://www.ensembl.org/id/ENST00000304494) [ENST00000579755](http://www.ensembl.org/id/ENST00000579755) [ENST00000361570](http://www.ensembl.org/id/ENST00000361570) |

1. MYC

| **Technology:** |  | qPCR |
| --- | --- | --- |
| **Assay Type:** |  | SYBR® Green |
| **Application:** |  | Gene Expression |
| **Unique Assay ID:** |  | qHsaCID0012921 |
| **Assay Design:** |  | Intron-spanning |
| **Chromosome Location:** |  | 8:128751241-128752749 |
| **Amplicon Length:** |  | 103 |
| **Splice Variants Targeted:** |  | [ENST00000377970](http://www.ensembl.org/id/ENST00000377970) [ENST00000524013](http://www.ensembl.org/id/ENST00000524013) [ENST00000454617](http://www.ensembl.org/id/ENST00000454617) |

1. TP53

| **Technology:** |  | qPCR |
| --- | --- | --- |
| **Assay Type:** |  | SYBR® Green |
| **Application:** |  | Gene Expression |
| **Unique Assay ID:** |  | qHsaCED0045022 |
| **Assay Design:** |  | exonic |
| **Chromosome Location:** |  | 17:7578471-7579324 |
| **Amplicon Length:** |  | 67 |

Splice variants targeted [ENST00000508793](http://www.ensembl.org/id/ENST00000508793) [ENST00000514944](http://www.ensembl.org/id/ENST00000514944) [ENST00000509690](http://www.ensembl.org/id/ENST00000509690)[ENST00000445888](http://www.ensembl.org/id/ENST00000445888) [ENST00000420246](http://www.ensembl.org/id/ENST00000420246) [ENST00000455263](http://www.ensembl.org/id/ENST00000455263)[ENST00000269305](http://www.ensembl.org/id/ENST00000269305) [ENST00000359597](http://www.ensembl.org/id/ENST00000359597) [ENST00000413465](http://www.ensembl.org/id/ENST00000413465)[ENST00000399121](http://www.ensembl.org/id/ENST00000399121) [ENST00000595022](http://www.ensembl.org/id/ENST00000595022) [ENST00000283365](http://www.ensembl.org/id/ENST00000283365)[ENST00000269190](http://www.ensembl.org/id/ENST00000269190) [ENST00000444659](http://www.ensembl.org/id/ENST00000444659) [ENST00000399097](http://www.ensembl.org/id/ENST00000399097)[ENST00000381801](http://www.ensembl.org/id/ENST00000381801) [ENST00000349699](http://www.ensembl.org/id/ENST00000349699) [ENST00000394367](http://www.ensembl.org/id/ENST00000394367)

1. TP73

| **Technology:** |  | qPCR |
| --- | --- | --- |
| **Assay Type:** |  | SYBR® Green |
| **Application:** |  | Gene Expression |
| **Unique Assay ID:** |  | qHsaCID0006518 |
| **Assay Design:** |  | Intron-spanning |
| **Chromosome Location:** |  | 1:3639949-3643754 |
| **Amplicon Length:** |  | 131 |

| **Splice Variants Targeted:** |  | [ENST00000378295](http://www.ensembl.org/id/ENST00000378295) [ENST00000354437](http://www.ensembl.org/id/ENST00000354437) [ENST00000378288](http://www.ensembl.org/id/ENST00000378288)[ENST00000378285](http://www.ensembl.org/id/ENST00000378285) [ENST00000378280](http://www.ensembl.org/id/ENST00000378280) [ENST00000357733](http://www.ensembl.org/id/ENST00000357733)[ENST00000346387](http://www.ensembl.org/id/ENST00000346387) [ENST00000378290](http://www.ensembl.org/id/ENST00000378290) |
| --- | --- | --- |
