# Supplementary material for: Loss of the proteasomal deubiquitinase USP14 induces growth defects and a senescence phenotype in colorectal cancer cells
Source: Sci Rep. 2024 Jun 6;14:13037. doi: 10.1038/s41598-024-63791-5 (PMC11156967; doi:10.1038/s41598-024-63791-5)
Supplement: Supplementary file 2 — Supplementary Information 2. [file 41598_2024_63791_MOESM2_ESM.pdf]

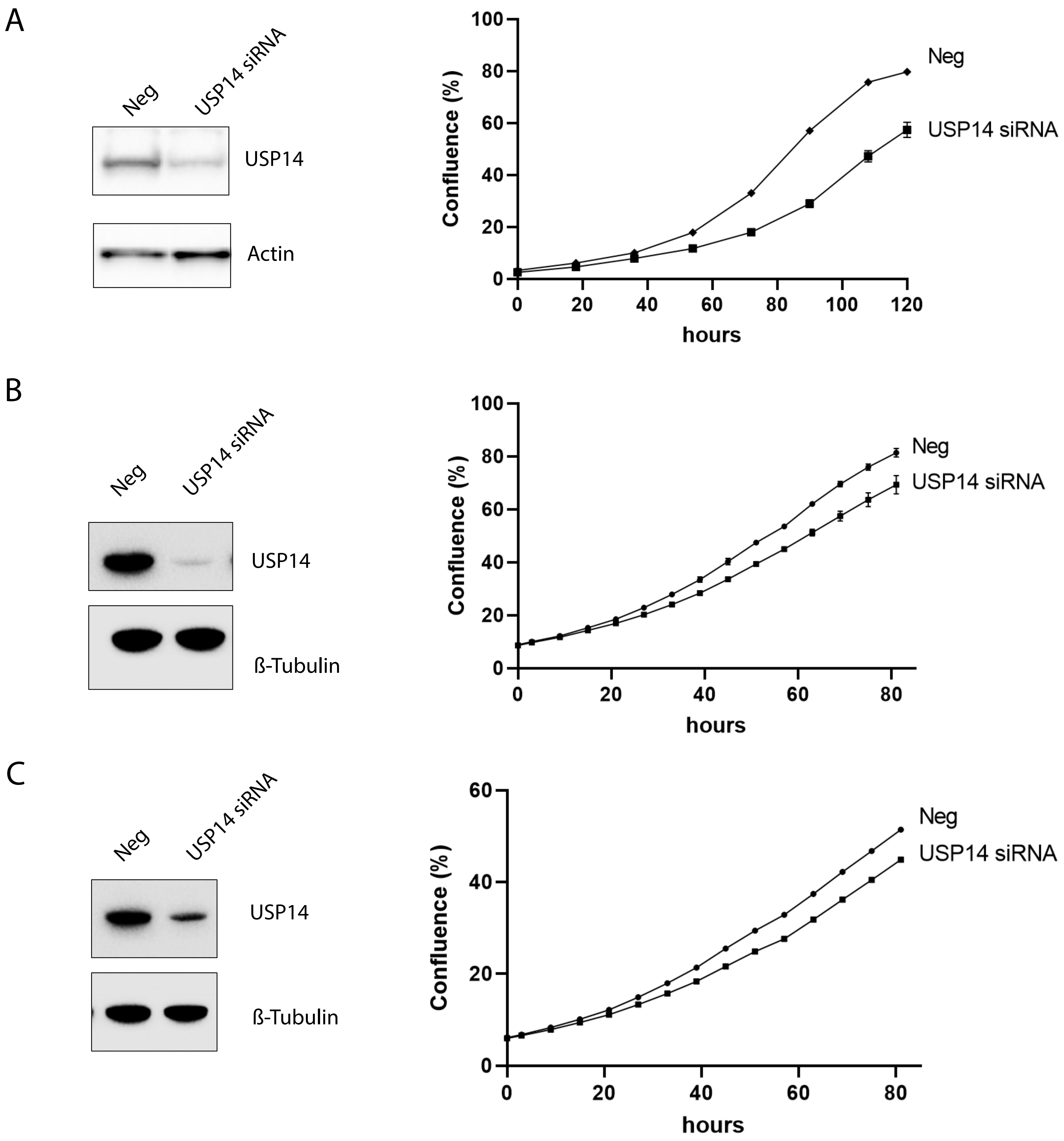

Fig S1. siRNA-induced inhibition of USP14 impairs cellular growth. Figures show immunoblots for USP14 72 hours after siRNA transfection and confluency based on live-cell imaging of A.) HCT116, B.) DLD1 cells, and C.) SW620 cells. Each data point is the average of three replicates +/- SD. Uncropped blots are shown in Fig S11.

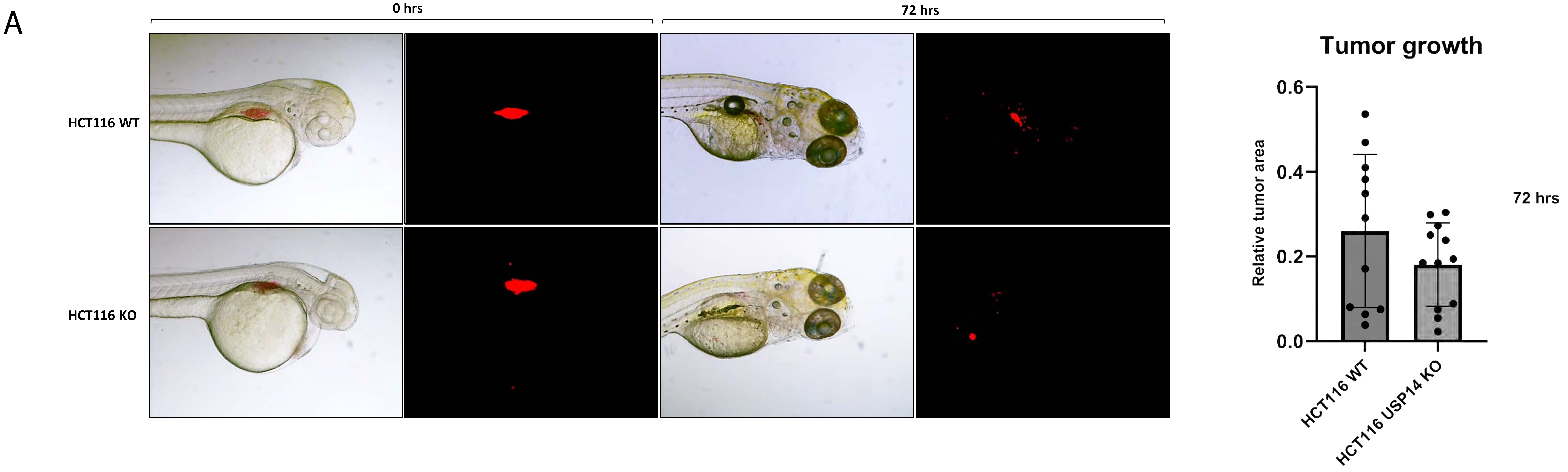

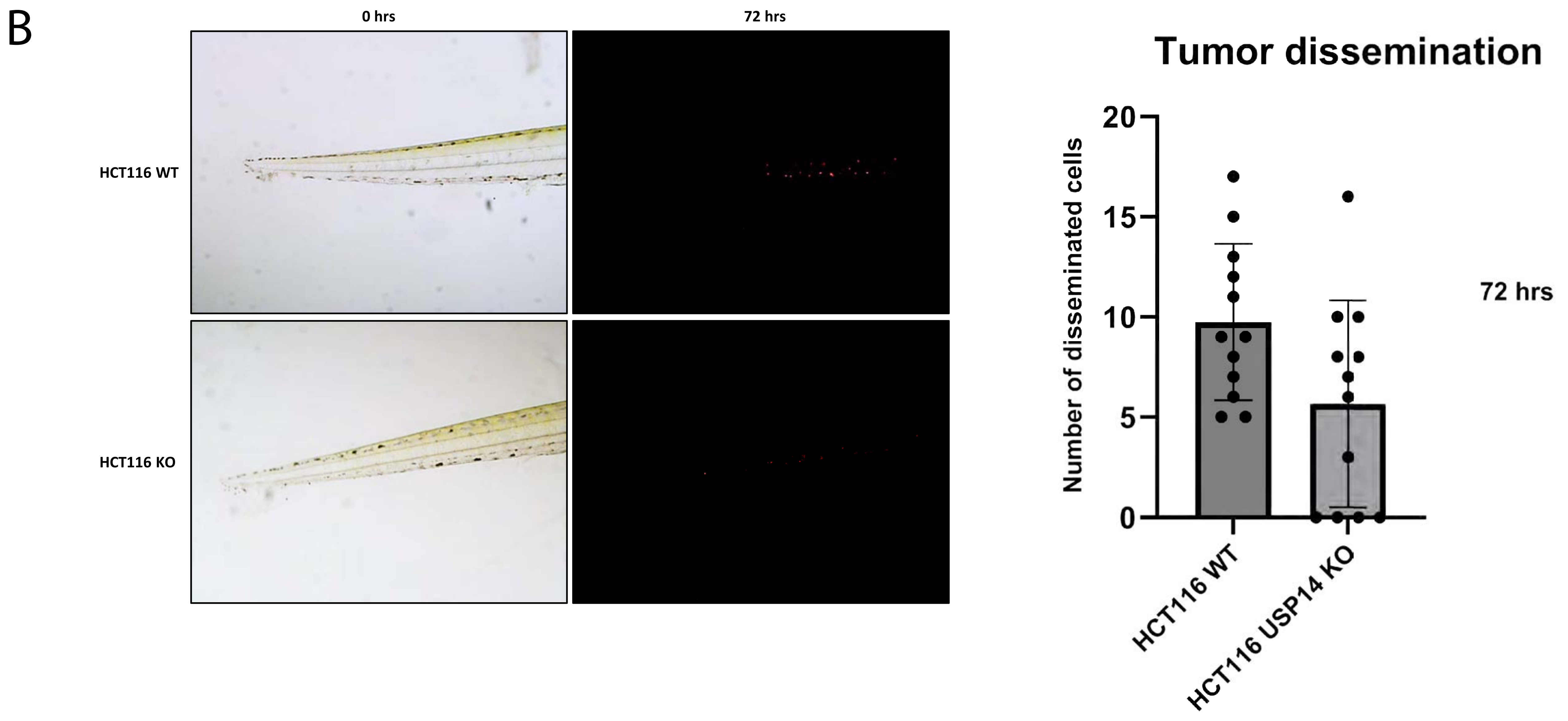

Fig S2. Effect of knocking out USP14 on growth and dissemination of HCT116 cells in a Zebrafish xenograft model . HCT116 cells were fluorescent-labeled and implanted into zebrafish embryos. Measurements were taken at 0 and 72h. A.) Tumor growth. Representative images of the tumor in the perivitelline space are shown. Columns show the average of the relative change in tumor sizes 72h after implantation +/- SD (p-value = 0.2) B.) Tumor dissemination. Representative images of the zebrafish tail showing disseminated tumors. Columns represent the average number of tumors found on the zebrafish tail 72h after implantation (p-value = 0.04) (n: WT=11, USP14-KO = 12)

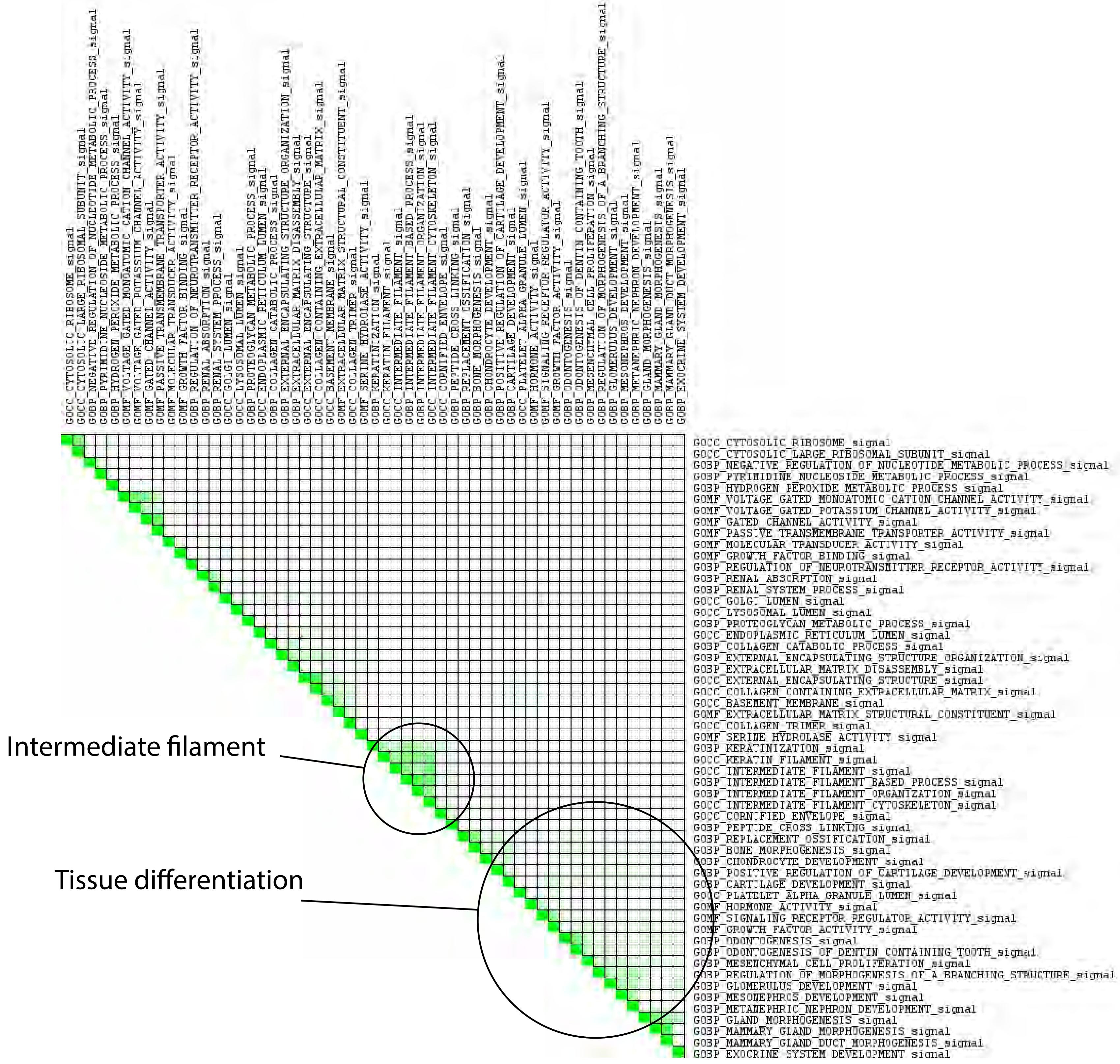

Fig S3. Leading Edge Analysis of enriched GO gene sets in upregulated genes. Circles highlight related genesets related to intermediate filaments and tissue differentiation

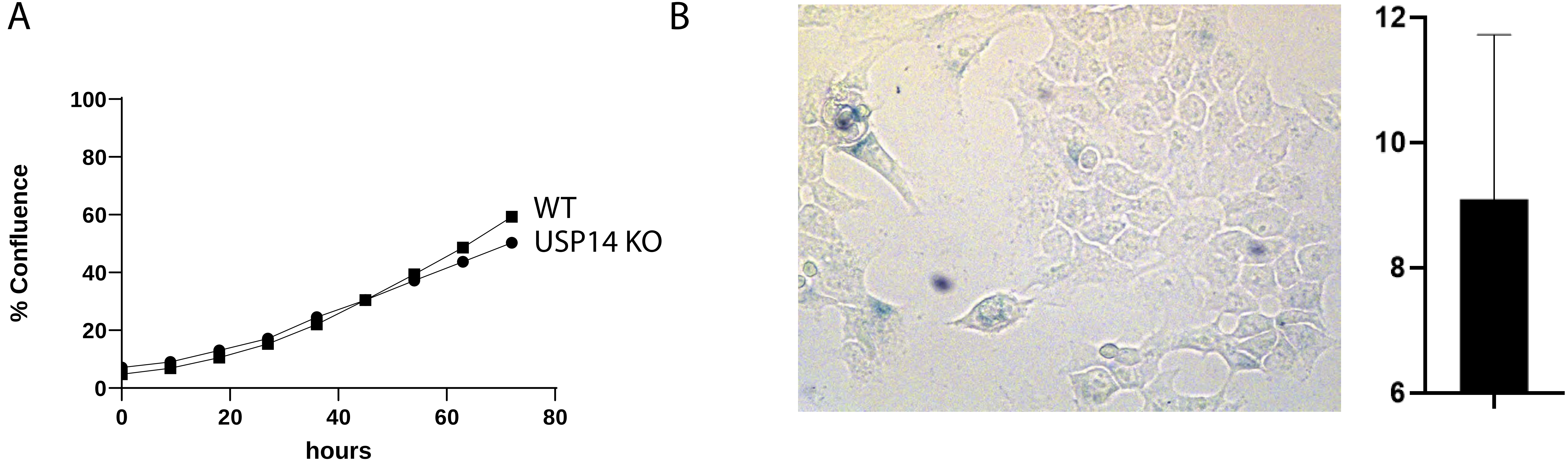

Fig S4. Growth and β-Gal staining of late-passage USP14 KO. A.) Confluence of USP14 KO cells vs WT on live-cell imaging at 22 passages after flow cytometric enrichment B.) β-Gal staining and % of β-Gal positive cells in microscopic slides

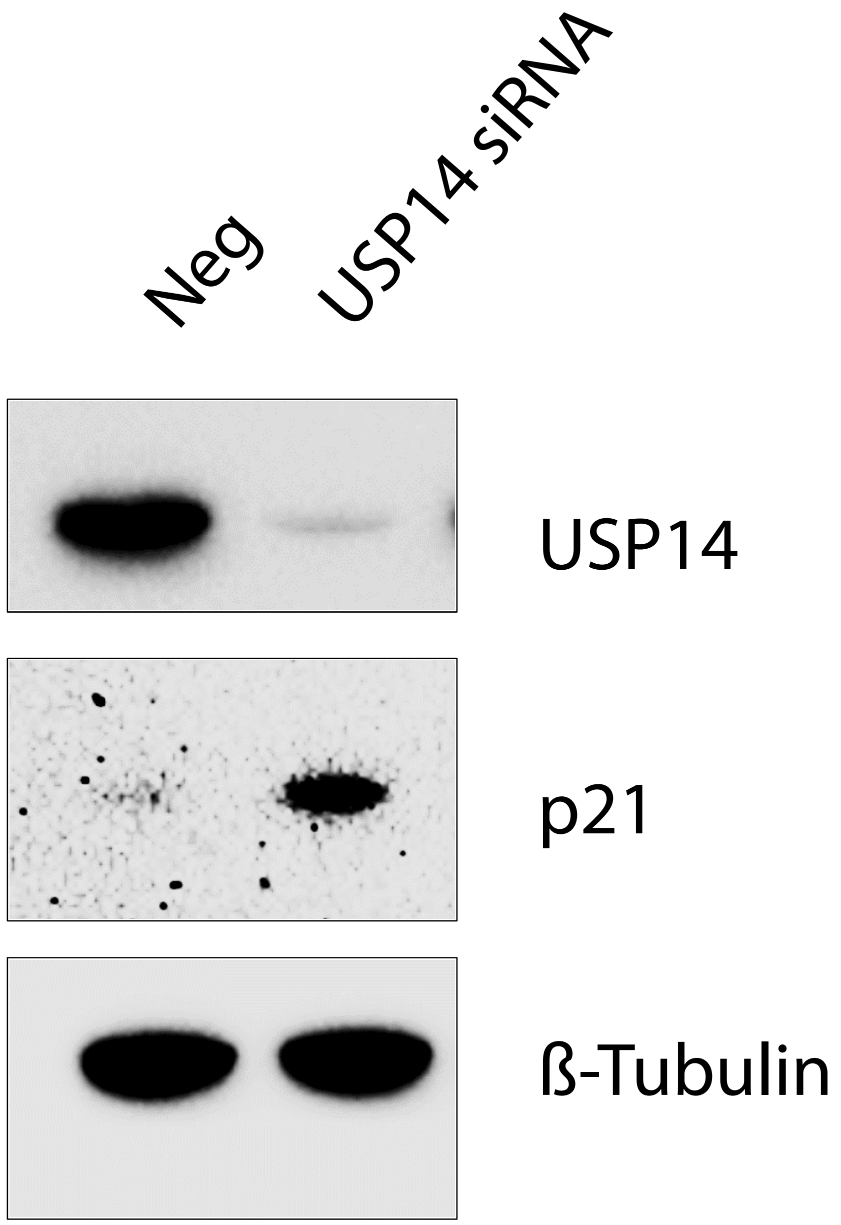

Fig S5. Immunoblot for p21 after siRNA-mediated knockdown of USP14 in DLD1 cells. Uncropped blots are shown in Fig S11

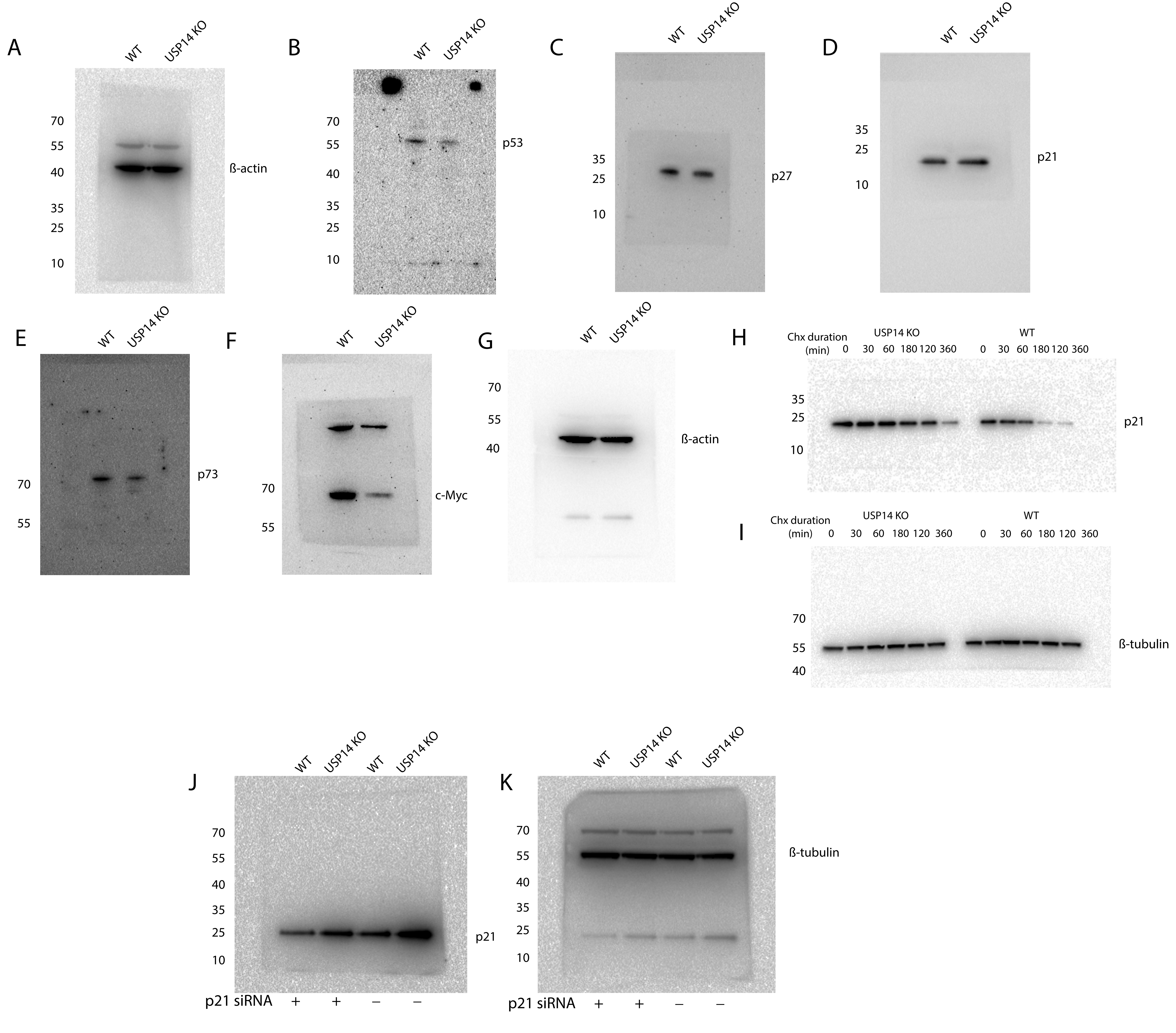

Fig S6. Full blots used in Fig 3. Blots A-G were used for Fig 3E. A and B were from the same blot, C- G were from the same blot. Samples used in these blots were the same and were loaded equally. For blots C - G, the blot was cut around the 40 kD mark. Blots H and I were used for Fig 3F. The images are from the same blot that was cut around the 40 kD mark. Blots J&K were used for Fig 3G

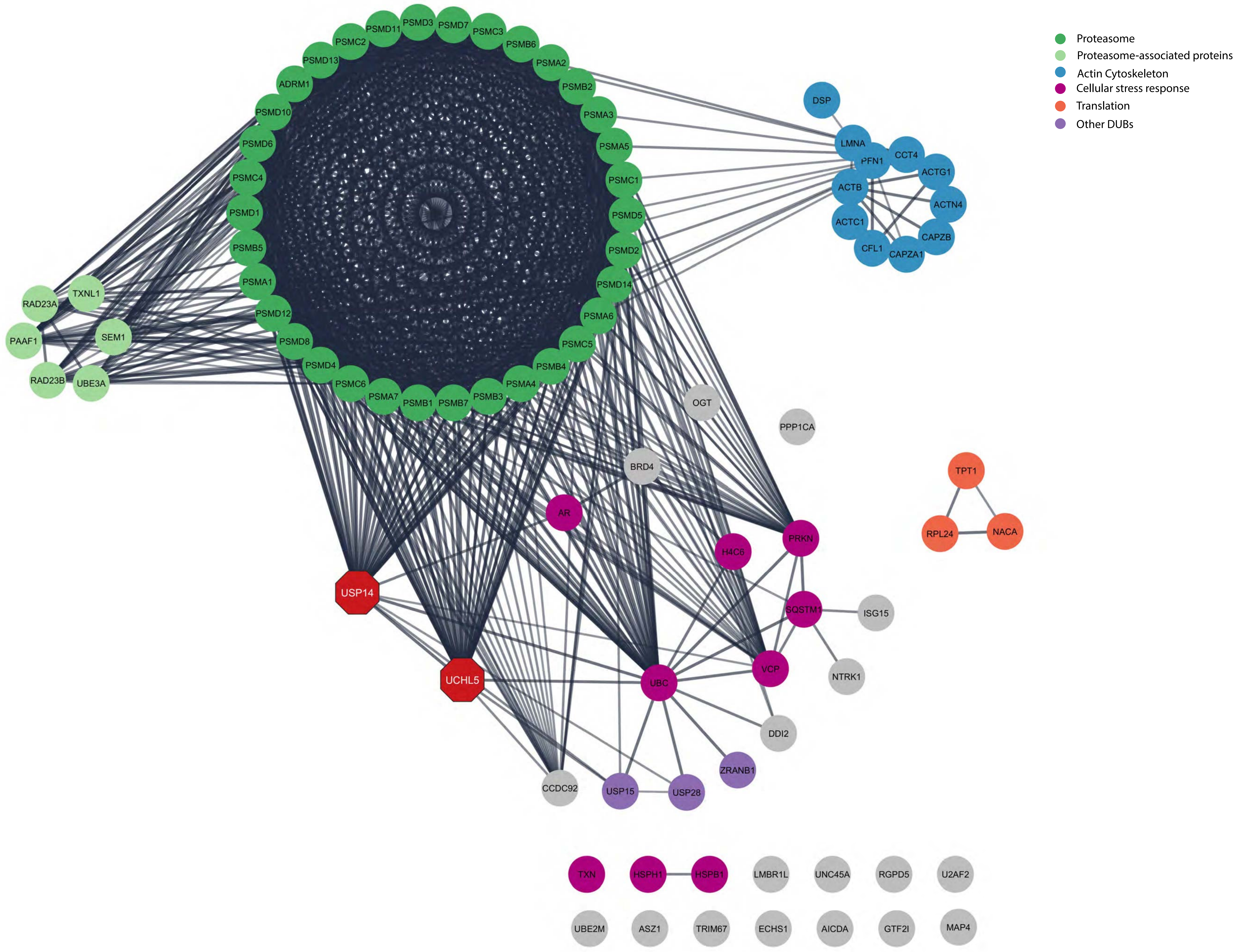

Fig S7. Network of commonly interacting proteins for UCHL5 and USP14. Edges are based on high confidence (0.700) functional or physical interactions based on strings.db

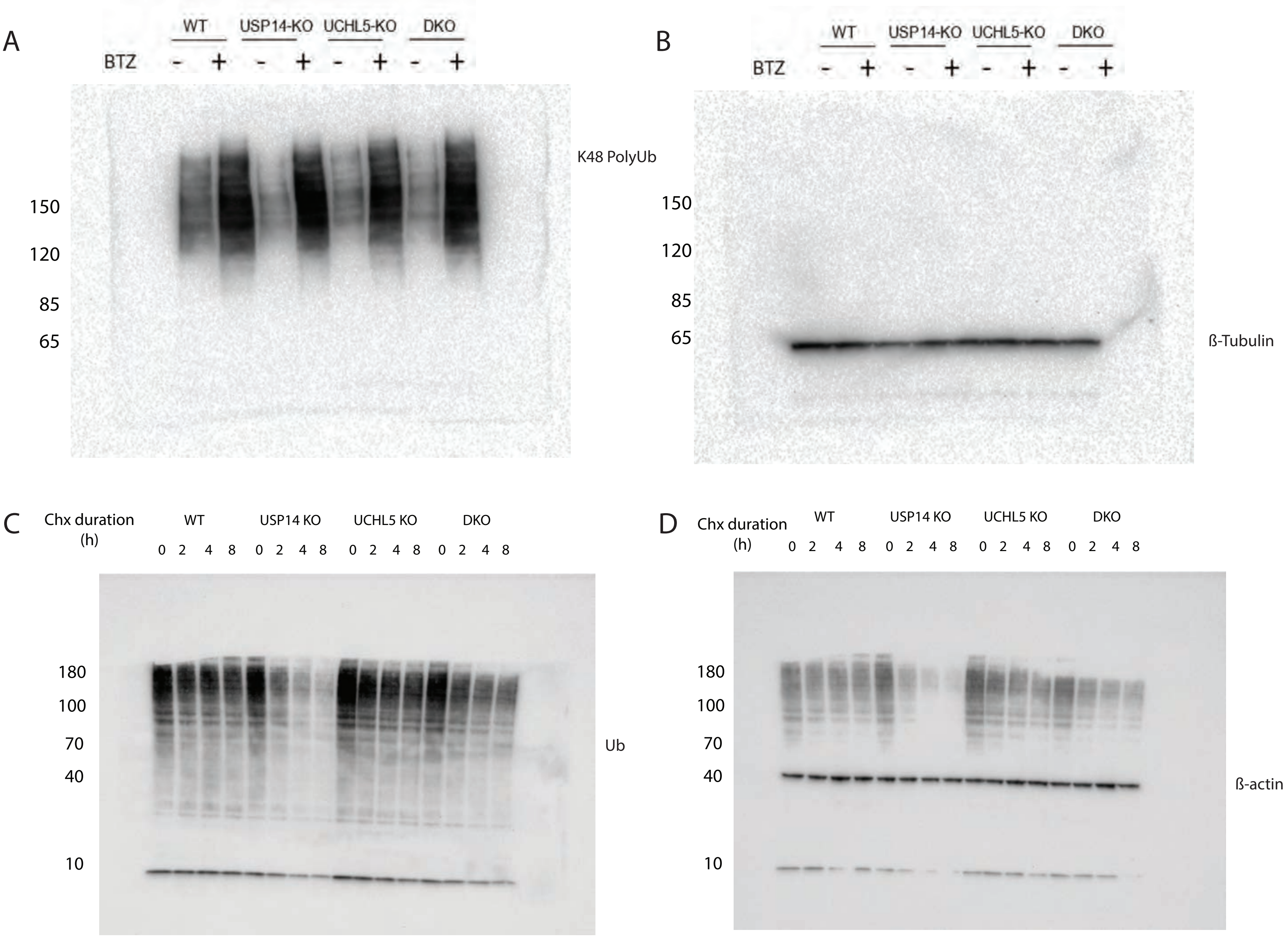

Fig S8. Full blots used in Fig 4. A and B were used in Fig 4C. C and D were used in Fig 4E

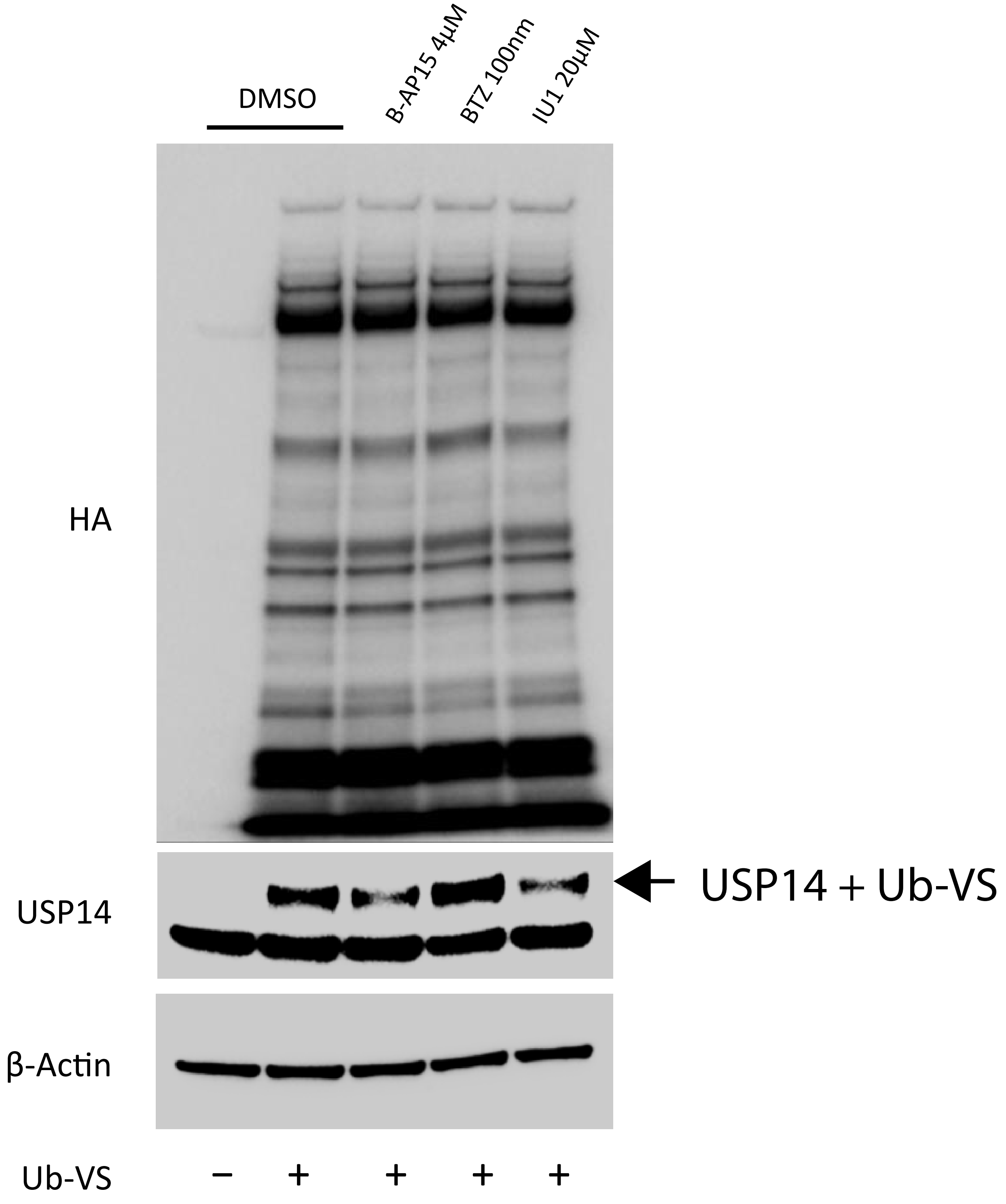

Fig S9. Activity based probe assay for USP14 with Ub-VS after treatment of the USP14 inhibitors b-AP15 and IU1, and the proteasome inhibitor bortezomib. A reduction in Ub-VS binding is observed only after b-AP15 and IU1 exposure.

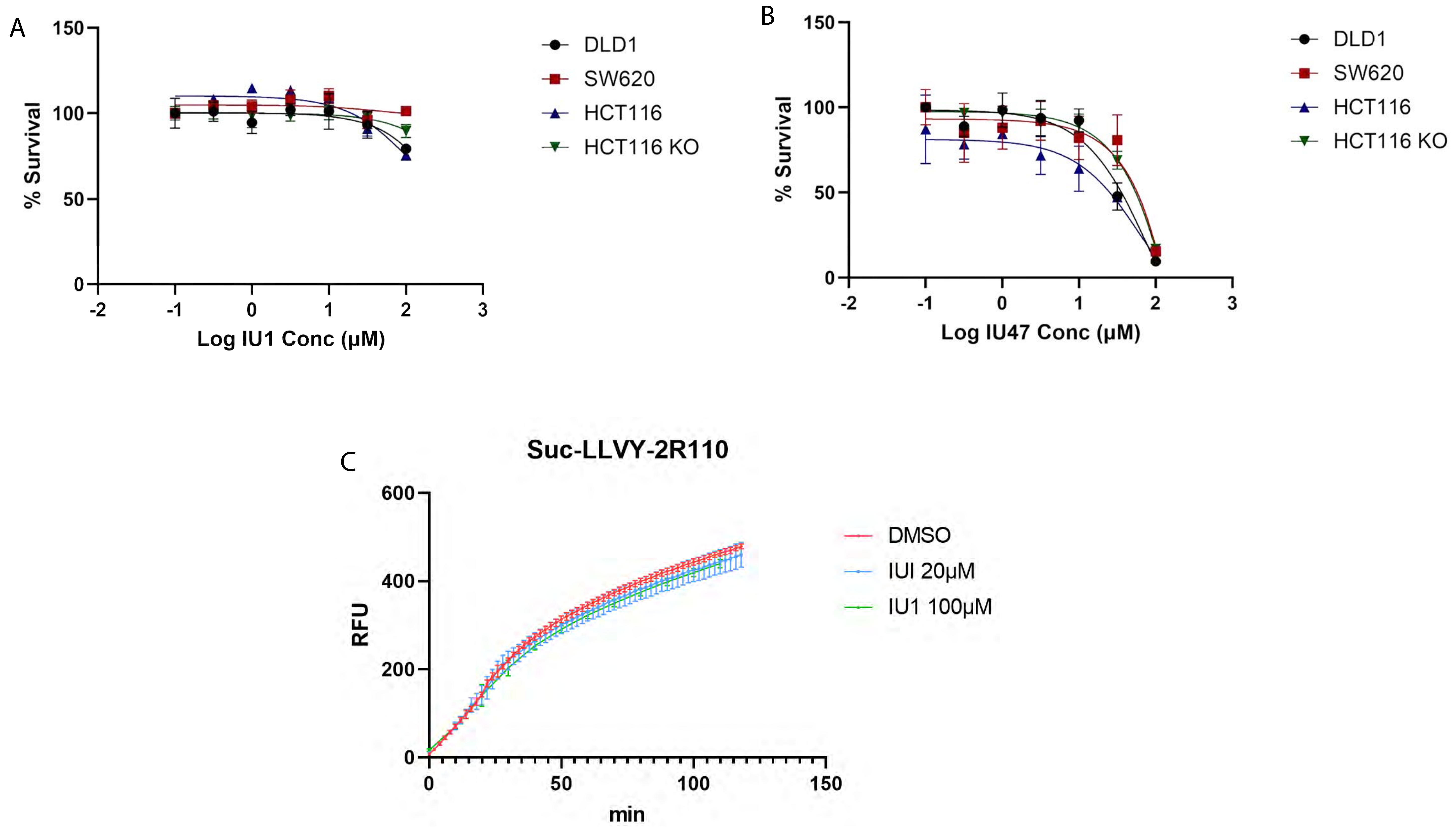

Fig S10. Effect of IU1 on cellular viability and proteasome proteolytic activity. Survival of colorectal cancer cell lines after 72-h exposure to 0.1-100µM IU1 (A) or IU1-47 (B). Survival is based on an MTT-assay. Each point is the average of three replicates ± SD. (C) Chymotrypsin-like activity of proteasome fractions purified from HEK293-Bio-Rpn11 cells containing DMSO, 20 or 100µM IU1were measured using the fluorogenic substrate Suc-LLVY-2R110. Each point represents the average of 4 replicates +/- SD.

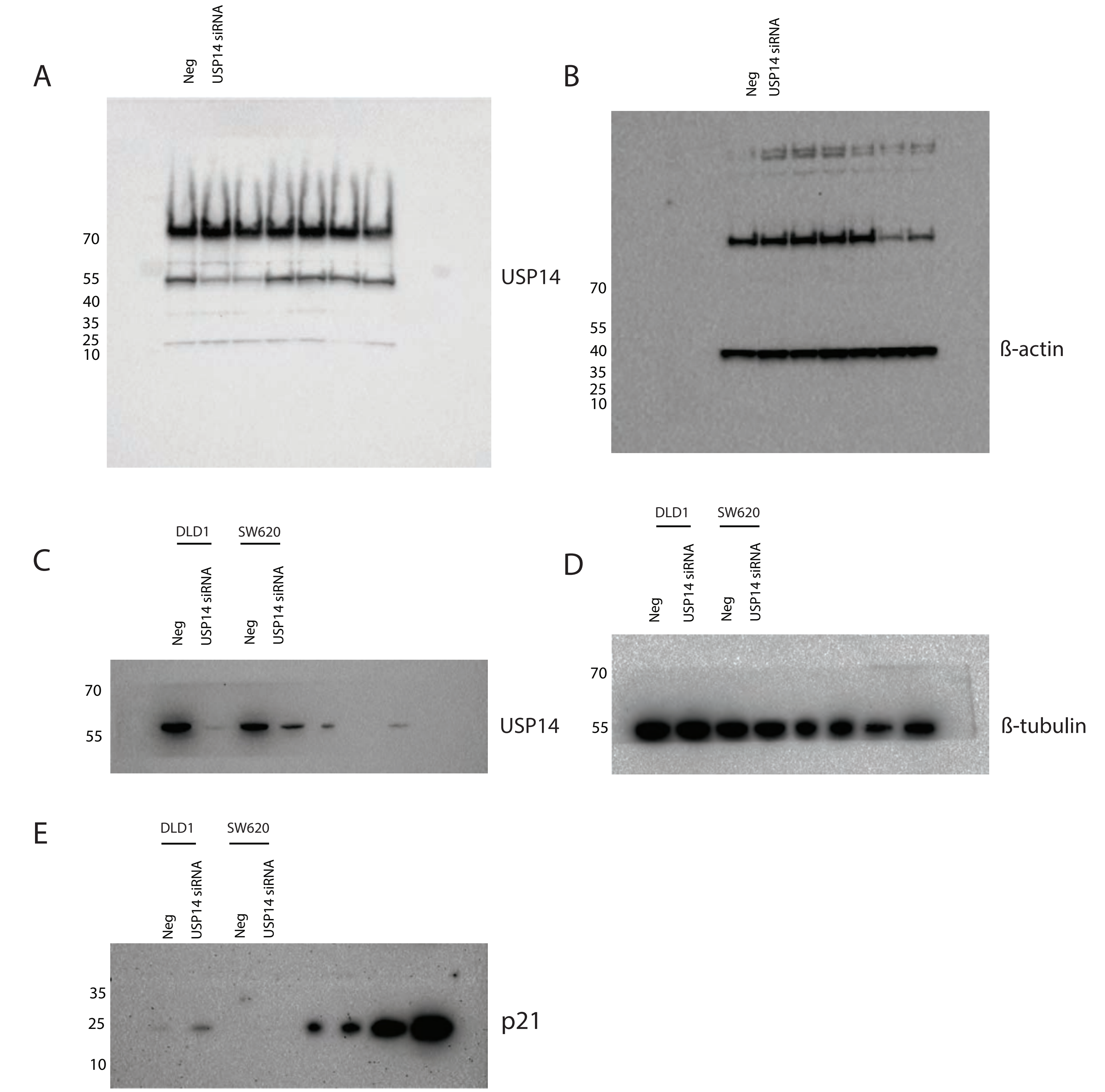

Fig S11. Full blots used in the supplementary section. A and B were used for S1A. Images are from the same blot. Unlabeled lanes are from other siRNA treatments not used in this study. C-E were used for the blots pertaining to DLD1 and SW620 in S1B, S1C and S5. Blots were cut at the 40kD mark. Unlabeled lanes are from protein samples not used in this study
