# Supplementary material for: Loss of the proteasomal deubiquitinase USP14 induces growth defects and a senescence phenotype in colorectal cancer cells
Source: Sci Rep. 2024 Jun 6;14:13037. doi: 10.1038/s41598-024-63791-5 (PMC11156967; doi:10.1038/s41598-024-63791-5)
Supplement: Supplementary file 7 — Supplementary Information 7. [file 41598_2024_63791_MOESM7_ESM.docx]

*Supplementary Methods*

**MTT Assay**

In each well of a 96-well plate, 10,000 cells were seeded. Varying concentrations of IU1 or IU1-47 between 0.1µM-100µM or vehicle were subsequently introduced. Following a 72-hour incubation period, 10 μl of MTT reagent (5 mg/ml) was added. After an additional 2-hour incubation, formazan crystals were dissolved by the addition 1 volume of 10%  SDS. Absorbance was quantified at 560 nm wavelength and analyzed using GraphPad software.

**Zebrafish Tumor Model**

HCT116 WT or KO cells were labeled with 1,10-dioctadecyl-3,3,303′-tetramethylindocarbocyanine (DiI), as previously described [[**1**](https://www.mdpi.com/2218-273X/11/9/1339#B18-biomolecules-11-01339)]. In brief, 70–80% confluent cells were washed with DPBS and incubated with DiI at a final concentration of 4 µg/mL for 30 min at 37 °C. After labeling, cells were washed twice in DPBS and kept on ice prior to implantation in zebrafish embryos. Transgenic Tg (fli1: EGFP)y1 zebrafish embryos were raised in E3-medium supplemented with PTU. Cells were resuspended at approximately 10^8^ cells per mL in cell growth medium, and approximately 400 cells in a 4 nL volume were implanted in the perivitelline space via sharp glass needles (world precision instruments, pulled in a PC-10 needle puller, Narishige, Tokyo, Japan) using a microinjection setup (MINJ-D, TriTech Research, Los Angeles, CA USA). Following injection, embryos were sorted for specific implantation of tumor cells in the perivitelline space and absence of cells in circulation under a fluorescent microscope (SMZ1500, Nikon, Tokyo, Japan) and placed in E3 embryo medium containing 0.2 mM PTU. Three days following tumor implantation, the embryos were anesthetized in MS-222 (0.04%, Sigma-Aldrich, St. Louis, MO, USA). Primary tumor sizes as well as the extent of local, peripheral, and hematogenous dissemination/metastasis of tumor cells were visualized under the fluorescent microscope. Results are shown as the average ± standard error of the mean of tumor volumes or number of cells present posterior to the anal opening. All animal experiments were approved by Linköpings Djurförsöksetiska Nämnd (N89/15).

**Effect of IU1 on proteasome chymotrypsin activity**

Proteasomes were isolated from HEK293-Bio-Rpn11 cells as previously described [2]. The isolated proteasomes, Suc-LLVY-2R110 (AAT-Bioquest), and the drug (IU1 or DMSO) were diluted separately in reaction buffer containing 50mM HEPES, 5mM MgCl2, 10% Glycerol v/v, 2mM ATP, and 1mM DTT. In a 384-well black opaque plate, Suc-LLVY-2R110 solution (working concentration of 50µM), drug solution (working concentration of DMSO, 20 or 100µM IU1), and proteasome solutions (working concentration 500pM) were added in sequence. Fluorescence was read using GloMax® Plate Reader (ProMega) at 3 min interval x 3 cycles under 37C at 496/520nm.

Reference:

1. Marques, I.J., Weiss, F.U., Vlecken, D.H., Nitsche, C., Bakkers J., Lagendijk, A.K., Partecke, L.I., Heidecke, C.D., Lerch, M.M., Bagowski, C.P. Metastatic behaviour of primary human tumours in a zebrafish xenotransplantation model. *BMC Cancer* 2009, *9*, 128.
2. Gubat, J., Selvaraju, K., Sjöstrand, L., Kumar Singh, D., Turkina, M.V., Schmierer, B., Sabatier, P., Zubarev, R.A., Linder, S., D'Arcy, P. Comprehensive Target Screening and Cellular Profiling of the Cancer-Active Compound b-AP15 Indicate Abrogation of Protein Homeostasis and Organelle Dysfunction as the Primary Mechanism of Action. Front Oncol. 2022 Apr 22;12:852980. doi: 10.3389/fonc.2022.852980.
