# Supplementary material for: Loss of the proteasomal deubiquitinase USP14 induces growth defects and a senescence phenotype in colorectal cancer cells
Source: Sci Rep. 2024 Jun 6;14:13037. doi: 10.1038/s41598-024-63791-5 (PMC11156967; doi:10.1038/s41598-024-63791-5)
Supplement: Supplementary file 8 — Supplementary Information 8. [file 41598_2024_63791_MOESM8_ESM.docx]

| Targets | Source | Catalog/Assay Number | Detected Transcripts |
| --- | --- | --- | --- |
| TP63 | Qiagen | Geneglobe ID: QT00024374 | NM_001114981, NM_001114980, NM_003722, NM_001114982, NM_001114978, NM_001114979, XM_005247846, XM_005247843, XM_005247844 |
| GAPDH | Qiagen | Geneglobe ID: QT00079247 | NM_002046, NM_001256799, NM_001289746, NM_001289745 |
| RRN18S | Qiagen | Geneglobe ID: QT00199367 | X03205 |
| UBB | Qiagen | Geneglobe ID: QT01009841 | NM_018955, NM_001281719, NM_001281718, NM_001281720, NM_001281716, NM_001281717 |
| UBC | Qiagen | Geneglobe ID: QT00234430 | XM_001132949, NM_021009 |
| RPS27a | Qiagen | Geneglobe ID: QT00002373 | NM_002954, NM_001177413, NM_001135592 |
| ACTB | Qiagen | Geneglobe ID: QT00095431w | NM_001101 |
| B2M | Qiagen | Geneglobe ID: QT00088935 | NM_004048, XM_006725182, XM_005254549 |
| CDKN1A | Biorad | qHsaCID0014498 | [ENST00000244741](http://www.ensembl.org/id/ENST00000244741)[, ENST00000373711](http://www.ensembl.org/id/ENST00000373711)[, ENST00000448526](http://www.ensembl.org/id/ENST00000448526)[, ENST00000405375](http://www.ensembl.org/id/ENST00000405375) |
| CDKN2A | Biorad | qHsaCED0056722 | [ENST00000304494](http://www.ensembl.org/id/ENST00000304494)[, ENST00000579755](http://www.ensembl.org/id/ENST00000579755)[, ENST00000361570](http://www.ensembl.org/id/ENST00000361570) |
| cMyc | Biorad | qHsaCID0012921 | [ENST00000377970](http://www.ensembl.org/id/ENST00000377970)[, ENST00000524013](http://www.ensembl.org/id/ENST00000524013) [, ENST00000454617](http://www.ensembl.org/id/ENST00000454617) |
| TP53 | Biorad | qHsaCED0045022 | [ENST00000508793](http://www.ensembl.org/id/ENST00000508793)[, ENST00000514944](http://www.ensembl.org/id/ENST00000514944)[, ENST00000509690](http://www.ensembl.org/id/ENST00000509690)[, ENST00000445888](http://www.ensembl.org/id/ENST00000445888)[, ENST00000420246](http://www.ensembl.org/id/ENST00000420246)[, ENST00000455263](http://www.ensembl.org/id/ENST00000455263)[, ENST00000269305](http://www.ensembl.org/id/ENST00000269305)[, ENST00000359597](http://www.ensembl.org/id/ENST00000359597)[, ENST00000413465](http://www.ensembl.org/id/ENST00000413465)[, ENST00000399121](http://www.ensembl.org/id/ENST00000399121)[, ENST00000595022](http://www.ensembl.org/id/ENST00000595022)[, ENST00000283365](http://www.ensembl.org/id/ENST00000283365)[, ENST00000269190](http://www.ensembl.org/id/ENST00000269190)[, ENST00000444659](http://www.ensembl.org/id/ENST00000444659)[, ENST00000399097](http://www.ensembl.org/id/ENST00000399097)[, ENST00000381801](http://www.ensembl.org/id/ENST00000381801)[, ENST00000349699](http://www.ensembl.org/id/ENST00000349699)[, ENST00000394367](http://www.ensembl.org/id/ENST00000394367) |
| TP73 | Biorad | qHsaCID0006518 | [ENST00000378295](http://www.ensembl.org/id/ENST00000378295), [ENST00000354437](http://www.ensembl.org/id/ENST00000354437), [ENST00000378288](http://www.ensembl.org/id/ENST00000378288), [ENST00000378285](http://www.ensembl.org/id/ENST00000378285), [ENST00000378280](http://www.ensembl.org/id/ENST00000378280), [ENST00000357733](http://www.ensembl.org/id/ENST00000357733), [ENST00000346387](http://www.ensembl.org/id/ENST00000346387), [ENST00000378290](http://www.ensembl.org/id/ENST00000378290) |

**Supplementary Table 1.** Primers used for qPCR

| Targets | Catalog Number |
| --- | --- |
| p21 | SI00604898 |
| USP14 | SI00072961 |
| USP14 | SI00072968 |
| Negative Control | SI03650318 |
| Positive (Cell Death) Control | SI04381048 |

**Supplementary Table 2.** siRNAs used. All were procured via Qiagen

| Target | Catalog Number | Source |
| --- | --- | --- |
| USP14 | A300-920A | Bethyl laboratories |
| c-Myc | 5605 | Cell Signaling Technology |
| p21 | 2946 | Cell Signaling Technology |
| p27 | 2552 | Cell Signaling Technology |
| p53 | 2527 | Cell Signaling Technology |
| p63 | 39692 | Cell Signaling Technology |
| p73 | 14620 | Cell Signaling Technology |
| Ubiquitin | Sc-8017 | Santa Cruz Biosciences |
| K48-linked Polyubiquitin | 05-1307 | Merck Millipore |
| Actin | A5316 | Sigma-Aldrich |
| Tubulin | T4026 | Sigma-Aldrich |
| Anti-Mouse IgG | 7076 | Cell Signaling Technology |
| Anti-Rabbit IgG | 7074 | Cell Signaling Technology |

**Supplementary Table 3.** Antibodies used for immunoblotting
